# Comparative efficacy and safety of Xuesaitong injection as adjuvant therapy for acute pancreatitis: a systematic review and meta-analysis

**DOI:** 10.3389/fphar.2026.1875983

**Published:** 2026-07-29

**Authors:** Yangbin Fang, Xin Chen, Luoyang Cai, Zhen Han, Zhengran Ma, Li Li, Zhenkun Gan, Tao Liu, Zheng Zuo, Yan Wang

**Affiliations:** 1 Yunnan University of Chinese Medicine, Kunming, Yunnan, China; 2 Zhejiang University of Chinese Medicine, Hangzhou, Zhejiang, China; 3 Dali Bai Autonomous Prefecture Chinese Medicine Hospital, Dali, Yunnan, China

**Keywords:** acute pancreatitis, meta-analysis, *Panax notoginseng* saponins, systematic review, Xuesaitong injection

## Abstract

**Background:**

Xuesaitong injection has been explored as an off-label adjuvant therapy for acute pancreatitis in China, but its efficacy and safety remain uncertain. This systematic review and meta-analysis evaluated Xuesaitong injection combined with conventional therapy for acute pancreatitis.

**Methods:**

Randomized controlled trials of Xuesaitong injection for acute pancreatitis were searched in the Cochrane Library, Embase, Web of Science, PubMed, China National Knowledge Infrastructure, Wanfang Database, VIP Database, and Chinese Biomedical Literature Database from inception to 1 March 2026. Two reviewers independently performed study screening, data extraction, and risk-of-bias assessment. Meta-analysis was conducted using a random-effects model, and certainty of evidence was assessed using the Grading of Recommendations Assessment, Development and Evaluation approach.

**Results:**

Sixteen randomized controlled trials involving 1,529 patients were included. Compared with conventional therapy alone, Xuesaitong injection plus conventional therapy improved the clinical response rate (relative risk = 1.15, 95% confidence interval 1.11–1.20), shortened length of hospital stay (mean difference = −5.18 days, 95% confidence interval −6.73 to −3.63), and reduced Acute Physiology and Chronic Health Evaluation II scores (mean difference = −1.99 points, 95% confidence interval −3.54 to −0.43). It also reduced inflammatory markers and amylase levels, including tumor necrosis factor-alpha, C-reactive protein, interleukin-6, interleukin-2, soluble intercellular adhesion molecule-1, serum amylase, and urinary amylase. The incidence of reported safety-related events was 6.43% in the experimental group and 7.04% in the control group. No serious adverse events clearly attributed to Xuesaitong injection were reported. Six studies had a high risk of bias, and the certainty of evidence was low or very low.

**Conclusion:**

Xuesaitong injection may provide additional benefits as adjuvant therapy for acute pancreatitis. However, methodological limitations, substantial heterogeneity, possible publication bias, and insufficient safety reporting warrant cautious interpretation. High-quality multicenter randomized controlled trials are needed.

**Systematic Review Registration:**

https://www.crd.york.ac.uk/PROSPERO/view/CRD420261335764, identifier CRD420261335764.

## 1 Introduction

Acute pancreatitis (AP) is an acute inflammatory disease of the pancreas caused by various etiologies. Clinically, it is mainly characterized by acute upper abdominal pain and may be accompanied by nausea, vomiting, and elevated serum amylase or lipase levels. It may further progress to local complications, such as peripancreatic fluid collections and pancreatic or peripancreatic necrosis, as well as systemic complications including organ dysfunction; severe cases may be life-threatening (Tenner et al., 2024). Clinically, acute pancreatitis is commonly classified as mild, moderately severe, or severe according to the presence and duration of organ dysfunction, particularly whether it persists for 48 h or longer, in combination with local and systemic complications (Banks et al., 2013). The clinical course of AP may be self-limiting in mild cases, but it can also rapidly progress to moderately severe or severe disease, thereby requiring continuous management from acute-phase treatment to recovery-phase care. Therefore, early assessment, stratified management, and prevention and control of complications are crucial to treatment (Trikudanathan et al., 2024).

At present, the burden of AP remains substantial both globally and in China. In the general population, the number of prevalent cases was estimated to be approximately 5.9 million worldwide and 488,000 in China in 2021 (Cai et al., 2025). In addition, a systematic review of time-trend studies showed that the incidence of AP has continued to increase in most Western countries, with an average annual percent change of approximately 3.07% in recent years; the corresponding figures were about 3.67% in North America and 2.77% in Europe, respectively (Iannuzzi et al., 2022). Although severe acute pancreatitis (SAP) accounts for only about 15%–20% of all cases of acute pancreatitis (AP), the risk of death is significantly higher; some studies suggest that its case-fatality rate reaches 20% (Oland and Hines, 2022). AP also imposes substantial healthcare resource use and economic burden. In the United States, it accounts for approximately 300,000 hospitalizations annually and generates more than USD 2 billion in healthcare expenditures (Lo et al., 2025). Therefore, further optimization of therapeutic strategies for patients with moderately severe or severe AP may help reduce the risk of complications and mortality, and is also of great importance for alleviating the burden on healthcare resources.

Currently, the management of AP is mainly based on supportive care, including early fluid resuscitation, analgesia, nutritional support, and correction of fluid, electrolyte, and acid–base disturbances, with anti-infective therapy and interventional drainage administered when necessary (Beij et al., 2025). These therapeutic measures play an important role in stabilizing vital signs and reducing the risk of complications; however, they remain limited in the management of patients with moderately severe or severe AP. In particular, specific and sustained pharmacological interventions targeting key pathological processes, such as excessive inflammatory responses, microcirculatory dysfunction, and organ injury, are still lacking, and some patients may still progress to persistent organ dysfunction or infectious complications (Leppäniemi et al., 2019). Moreover, intensive treatment depends heavily on multidisciplinary collaboration and resource availability, and variations in therapeutic strategies across centers may also affect patient outcomes (Hamesch et al., 2025). Therefore, how to further improve disease severity and key clinical outcomes while ensuring safety remains a practical challenge.

In China, Xuesaitong injection has attracted attention as an adjuvant therapeutic option. Its main active constituents are *Panax notoginseng* saponins, which are extracted from the traditional Chinese medicine *Panax notoginseng* (Liu et al., 2026). Previous studies have suggested that Xuesaitong injection may improve microcirculatory perfusion and endothelial function (Zhang et al., 2026), regulate inflammation-related pathways such as nuclear factor-kappa B (NF-κB) and mitogen-activated protein kinase (MAPK) (Kim et al., 2025), and exert antiplatelet and antithrombotic effects (Wu Y. et al., 2022). These pharmacological actions may help attenuate systemic inflammatory responses and organ injury, thereby improving indicators of disease severity and inflammation-related parameters. At present, Xuesaitong injection is mainly approved in China for cardio-cerebrovascular and ophthalmic vascular diseases, including post-stroke hemiplegia, atherosclerotic cerebral infarction, cerebral embolism, and central retinal vein occlusion. Its use in acute pancreatitis should therefore be interpreted as an off-label exploratory adjuvant application rather than an established standard indication. However, in recent years, several clinical studies have explored this off-label application in the treatment of acute pancreatitis, suggesting a potential therapeutic role that may be related to its anti-inflammatory activity, microcirculation-improving effects, and vascular endothelial protection (Li X. et al., 2023). In addition, the Chinese expert consensus on the clinical application of *Panax notoginseng* saponin preparations lists these preparations as a potential adjuvant therapeutic option for acute pancreatitis (National Clinical Research Center for Chinese Medicine Cardiology et al., 2021). Xuesaitong injection is widely used in clinical practice and has a clearly defined route of administration. As a supplementary option within comprehensive treatment, it has certain accessibility and generalizability, and may therefore serve as a useful adjunct to the integrated management of AP (He et al., 2020).

Although several randomized controlled trials have evaluated the adjuvant efficacy of Xuesaitong injection in acute pancreatitis, the current evidence remains insufficient. Differences across studies in the included populations, intervention regimens, comparator treatments, follow-up duration, and outcome definitions have led to inconsistent findings; moreover, some studies were limited by small sample sizes, single-center designs, and insufficient reporting of key methodological details, such as randomization and blinding, resulting in potential risks of bias and making it difficult for individual studies to draw robust conclusions. To date, there remains a lack of methodologically rigorous systematic reviews and meta-analyses specifically evaluating Xuesaitong injection for the treatment of acute pancreatitis with consistently focused outcomes.

Therefore, we conducted this systematic review and meta-analysis to systematically synthesize the available evidence from clinical controlled studies and to evaluate the efficacy and safety of Xuesaitong injection in patients with acute pancreatitis, with particular emphasis on its effects on inflammation-related indicators and severity-related outcomes. We used a random-effects model for pooled analyses and prespecified subgroup and sensitivity analyses to assess the robustness of the findings and explore potential sources of heterogeneity. Publication bias was also evaluated, and the certainty of evidence was comprehensively assessed using an evidence grading approach, with the aim of providing an evidence-based basis for clinical decision-making and future high-quality research.

## 2 Materials and methods

### 2.1 Description of the intervention

This study was conducted with reference to the four core pillars of best practice in ethnopharmacology, namely, the traceability of botanical sources and pharmacognostic characteristics, the rationale for pharmacological relevance, the assessability of clinical medication safety, and the relevance to clinical and therapeutic contexts. Xuesaitong injection is approved by the National Medical Products Administration of China and is manufactured in accordance with the Chinese national drug standard WS3-B-3590-2001 (Z)-2011. According to the package insert, its indicated uses include post-stroke hemiplegia, atherosclerotic thrombotic cerebral infarction, cerebral embolism, and central retinal vein occlusion.

Quality control procedures for Xuesaitong injection involve the identification and quantification of key bioactive constituents, including notoginsenoside R1, ginsenoside Rg1, ginsenoside Re, ginsenoside Rb1, and ginsenoside Rd. These procedures, together with chromatographic fingerprinting and content determination, allow comprehensive evaluation of its chemical consistency and quality stability. Previous reviews have indicated that Xuesaitong injection has a favorable safety profile, with no major adverse events reported.

All botanical names were validated using the Medicinal Plant Names Services (MPNS) database. Pharmacopoeial drug names and standards were confirmed against Pharmacopoeia of the People’s Republic of China (2020 Edition) (Table 1).

**TABLE 1:** Taxonomic and pharmacopoeial details of the botanical drug used in Xuesaitong injection.

Chinese drug name	Botanical name (with authority)	Family	Pharmacopoeial drug name
Sanqi	*Panax notoginseng* (Burkill) F. H. Chen	Araliaceae	Notoginseng Radix et Rhizoma

### 2.2 Study profile

The study protocol was prospectively registered on the PROSPERO platform (registration number: CRD420261335764). This systematic review was conducted and reported in strict accordance with the 2020 Preferred Reporting Items for Systematic Reviews and Meta-Analyses (PRISMA) guidelines (Page et al., 2021).

### 2.3 Eligibility criteria according to the population, intervention, comparator, and outcomes (PICO) framework

#### 2.3.1 Population (P)

Randomized controlled trials involving patients aged 18 years or older who were diagnosed with acute pancreatitis were eligible for inclusion. The diagnosis of acute pancreatitis was required to conform to recognized guidelines or consensus criteria and was generally defined by the presence of at least two of the following three features: typical clinical manifestations consistent with acute pancreatitis, such as acute upper abdominal pain; serum amylase and/or lipase levels elevated to at least three times the upper limit of normal; and characteristic imaging findings suggestive of acute pancreatitis. Studies that reported only chronic pancreatitis or acute exacerbation of chronic pancreatitis, without allowing these patients to be distinguished from those with acute pancreatitis, were excluded. Studies involving patients with elevated pancreatic enzymes or abdominal pain clearly attributable to non-pancreatitis causes and not meeting the above diagnostic criteria were also excluded.

#### 2.3.2 Intervention (I)

Eligible studies were required to administer Xuesaitong injection as an adjuvant intervention in addition to conventional therapy. No prespecified restrictions were imposed on the dosage, frequency, or treatment duration of Xuesaitong injection, in order to capture variations in real-world clinical practice as far as possible and improve the generalizability of the synthesized evidence. As Xuesaitong injection is a standardized traditional Chinese medicine injection, with consistent dosage form and quality standards under the same regulatory approval system, trials were included if they explicitly reported the use of Xuesaitong injection administered intravenously, even when detailed compositional information was not provided in each individual study. To avoid uncertainty in attributing treatment effects caused by concomitant medications, studies in which the intervention group also received other traditional Chinese medicine injections, or in which multiple traditional Chinese medicine injections were used in combination and the independent effect of Xuesaitong injection could not be isolated, were excluded.

#### 2.3.3 Comparator (C)

Eligible studies were required to include a comparable control group to evaluate the difference between Xuesaitong injection combined with conventional therapy and conventional therapy alone. Conventional therapy referred to basic supportive care and complication management for acute pancreatitis in accordance with guideline or consensus recommendations. This could include fasting and gastrointestinal decompression, fluid replacement and volume management, analgesic and antispasmodic treatment, nutritional support, correction of electrolyte and acid–base disturbances, anti-infective therapy when clearly indicated, and comprehensive supportive treatment for organ dysfunction. To ensure clear attribution of treatment effects, studies directly comparing Xuesaitong injection combined with one type of conventional therapy against a different conventional therapy were excluded. Studies in which the baseline treatment regimens differed between groups and such differences could potentially dominate outcome changes were also excluded.

#### 2.3.4 Outcomes (O)

The primary outcomes were the clinical response rate and the incidence of adverse events. The clinical response rate was selected as a primary outcome because relief of clinical symptoms and overall improvement in disease condition are among the most frequently reported outcomes in the treatment of acute pancreatitis and can be readily extracted across studies. This outcome may directly reflect the potential benefit of Xuesaitong injection on overall patient prognosis in most trials. The incidence of adverse events was also designated as a primary outcome to systematically evaluate the safety profile of Xuesaitong injection. Given the priority of safety assessment in the clinical use of traditional Chinese medicine injections, particularly in patients with acute diseases, including adverse events as a primary outcome may facilitate a more comprehensive evaluation of the risk–benefit balance.

Secondary outcomes included hospitalization-related outcomes and objective physiological and biochemical indicators. Hospitalization-related outcomes mainly included length of hospital stay and severity-related scores, which were included in the analysis when extractable from the original studies. Objective indicators included pancreatic injury-related markers, such as serum amylase and urinary amylase, inflammatory response markers, such as C-reactive protein and interleukins, as well as cytokine-related indicators, such as tumor necrosis factor. These secondary outcomes were used to reflect intermediate physiological effects and potential mechanisms of action. When primary outcomes were not reported or were insufficiently reported, they could provide supplementary surrogate information for clinical interpretation and support discussion of the possible mechanisms.

### 2.4 Literature search

This study systematically searched relevant studies in electronic databases, including the Cochrane Library, Embase, Web of Science, PubMed, China National Knowledge Infrastructure, Wanfang Data, VIP, and the China Biomedical Literature Database. The search period extended from the inception of each database to 1 March 2026. The search terms consisted of intervention-related and disease-related terms. Intervention-related terms were mainly developed around Xuesaitong injection and its common expressions, whereas disease-related terms focused on acute pancreatitis and its corresponding English terminology. The complete search strategies and search fields for each database are provided in Supplementary Material S1.

To minimize potential omissions, we also manually screened the reference lists of the included studies and relevant reviews to identify potentially missed original studies. All records retrieved from the searches were imported into EndNote X9.3.3 for reference management and deduplication. After duplicates were removed, the remaining records proceeded to the subsequent study screening process.

### 2.5 Screening and data extraction

Two reviewers independently performed study screening and data extraction according to the predefined inclusion and exclusion criteria. During the screening process, titles and abstracts were first reviewed to exclude records that were clearly irrelevant to the research topic. Subsequently, the full texts of potentially eligible studies were obtained and further assessed to confirm that the study population, intervention, comparator, outcomes, and study design met the predefined eligibility criteria before inclusion.

Data extraction was performed independently by two reviewers using a pre-designed data extraction form, and the extracted data were cross-checked. The extracted information included the first author, publication year, sample size, patient age and baseline characteristics, details of the intervention and control regimens such as dosage and treatment duration, raw outcome data, and key methodological elements required for risk-of-bias assessment. For incomplete or ambiguous data, efforts were made to contact the original authors to obtain additional information or clarification. Any disagreements during the screening or extraction process were first resolved through discussion between the two reviewers; if consensus could not be reached, a third reviewer was consulted for arbitration.

### 2.6 Risk of bias assessment

The risk of bias for each included study was independently assessed by two reviewers using the Cochrane Risk of Bias tool, version 2 (RoB 2.0) (Sterne et al., 2019). This tool covers five domains of potential bias, namely, bias arising from the randomization process, bias due to deviations from the intended interventions, bias in outcome measurement, bias due to missing outcome data, and bias due to selective reporting of results. Each domain was judged as low risk, some concerns, or high risk according to the criteria of the tool.

The overall risk of bias was determined according to predefined rules. A study was judged as having an overall low risk of bias only when all domains were rated as low risk. If any domain was judged as having some concerns, the overall risk of bias was accordingly rated as some concerns. If any domain was judged as high risk, the overall risk of bias was rated as high risk. After the two reviewers completed the independent assessments, the results were cross-checked. Any disagreements were first resolved through discussion, and if consensus could not be reached, a third reviewer was consulted for arbitration.

### 2.7 Statistical analysis

Meta-analysis was performed to quantitatively synthesize outcome data from the included randomized controlled trials. For dichotomous outcomes, relative risk (RR) with 95% confidence interval (CI) was used as the effect measure, and pooled estimates were calculated using the Mantel–Haenszel method. For continuous outcomes, the mean difference (MD) with 95% CI was preferentially used as the effect measure when measurement units were consistent, and pooled estimates were calculated using the inverse variance method. When different scales were used across studies or reporting units could not be standardized, the standardized mean difference (SMD) was applied to improve comparability.

To assess the robustness of the pooled results, sensitivity analyses were prespecified by sequentially excluding individual studies and by excluding studies judged to have an overall high risk of bias. Changes in the direction and statistical significance of the pooled effects were examined to determine the extent to which the conclusions depended on individual studies or risk of bias. Statistical heterogeneity was assessed using Cochran’s Q test and the I^2^ statistic. A random-effects model was used for all outcomes, regardless of the magnitude of heterogeneity. For outcomes showing substantial heterogeneity, prespecified subgroup analyses were conducted to explore potential sources of heterogeneity by stratifying studies according to treatment duration of ≤7 days or >7 days and mean age of ≥65 years or <65 years. For outcomes involving 10 or more studies, funnel plots combined with Harbord’s or Egger’s test were used to assess the possibility of publication bias. All statistical analyses were performed using RevMan software (version 5.4) for the main pooled analyses and graphical outputs, with R software (version 4.3.3) used for supplementary statistical tests when necessary.

### 2.8 Trial sequential analysis (TSA)

TSA was performed using the official TSA software (TSA, version 0.9.5.10 Beta; Copenhagen Trial Unit, Centre for Clinical Intervention Research, Denmark) to evaluate the risk of random errors and the robustness of cumulative evidence. A two-sided type I error of 5% and a power of 80% were applied. For the binary outcome of clinical response rate, the effect measure was risk ratio (RR), and the outcome was defined as beneficial, with an anticipated relative risk increase of 15%. For continuous outcomes, mean difference (MD) was used when outcomes were measured on the same scale, and the direction of effect was harmonized so that lower values favored the experimental group. The anticipated intervention effect and variance for continuous outcomes were estimated from the included trials in the TSA software. The diversity adjustment (D2) was set according to the heterogeneity observed in the corresponding meta-analysis. Trial sequential monitoring boundaries were constructed using an O'Brien-Fleming alpha-spending function under a random-effects model. TSA was conducted separately for the main outcomes with sufficient data.

### 2.9 Quality of evidence appraisal

As the included studies were randomized controlled trials, the initial certainty of evidence was rated as high. The certainty of evidence was then downgraded according to five core domains: risk of bias, inconsistency, indirectness, imprecision, and publication bias. When clear limitations were identified in any of these domains, the certainty of evidence was downgraded accordingly and ultimately classified as moderate, low, or very low, reflecting the credibility and stability of the conclusions (Balshem et al., 2011).

## 3 Results

### 3.1 Results of the literature search

**FIGURE 1:**
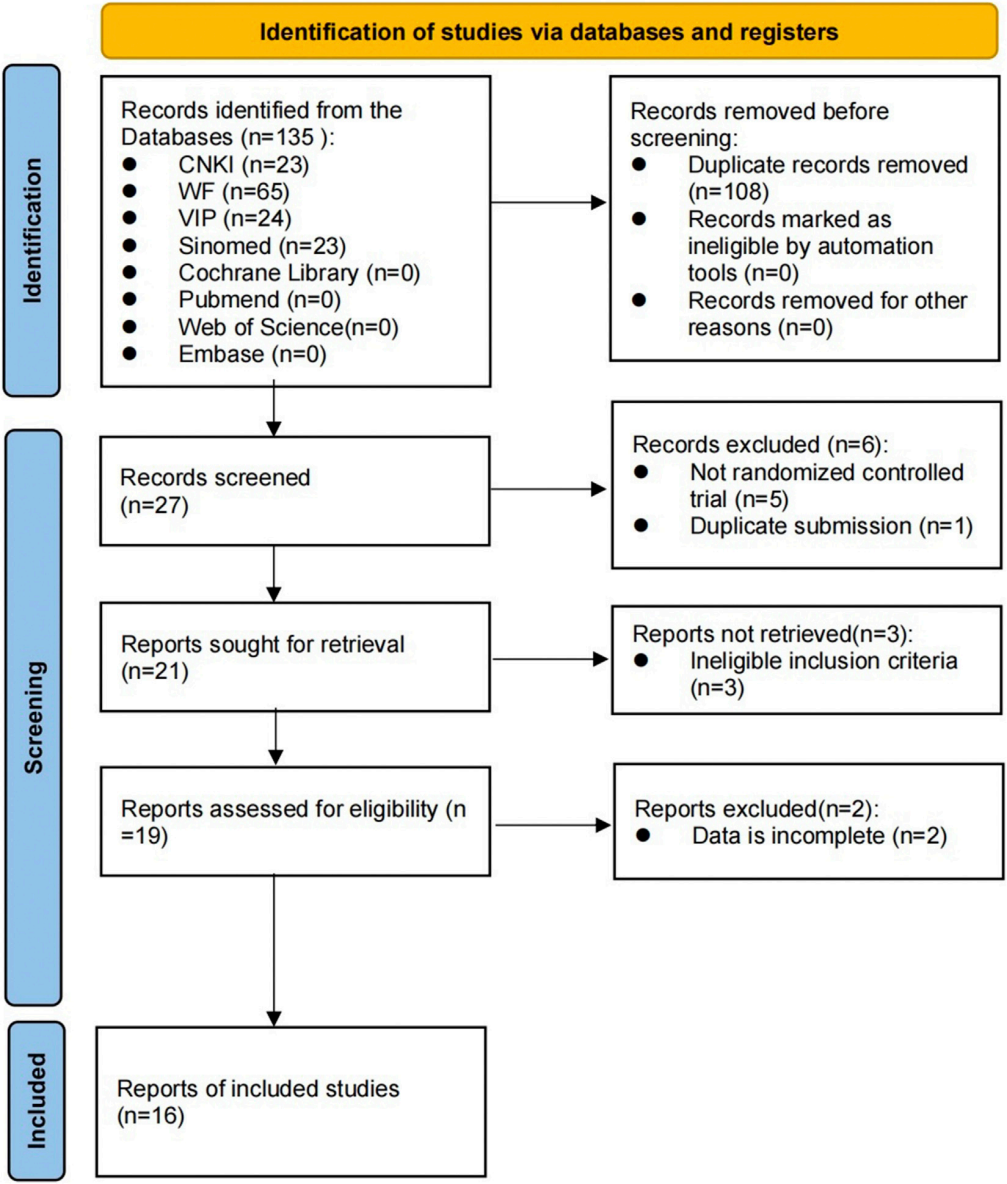
Process of literature screening.

A total of 135 records were initially identified through systematic searches, including 23 from China National Knowledge Infrastructure, 65 from Wanfang Data, 24 from VIP, 23 from Sinomed and none from the Cochrane Library, Embase, Web of Science, or PubMed. After deduplication, 108 duplicate records were removed, leaving 27 records for title and abstract screening. Six records were excluded at this stage, including five non-randomized controlled studies and one duplicate publication. Subsequently, 21 reports were retrieved for full-text assessment and further evaluation, of which two were excluded because they did not meet the eligibility criteria. After eligibility assessment of the remaining 18 full-text articles, two additional studies were excluded because of incomplete data. Ultimately, 16 studies were included in the qualitative and quantitative syntheses (Liu and Lu, 2004; Liu et al., 2006; Liu et al., 2024; Niu, 2008; Du, 2009; Li et al., 2016; Fang et al., 2017; Shi, 2018; Wang, 2018; Zhang, 2018; Zhang, 2019; Zhang, 2020; Yu, 2019; Lin, 2020; Song et al., 2021; Zou, 2021) (Figure 1).

### 3.2 Characteristics of the included studies

A total of 16 randomized controlled trials (RCTs) were included in this study. The publication years ranged from 2004 to 2024, and all studies were conducted in mainland China. The sample sizes of the included studies ranged from 60 to 240 patients, with generally balanced sample distributions between the experimental and control groups. The study populations mainly consisted of patients with acute pancreatitis, while some studies further restricted inclusion to patients with severe disease, older adults, or those with concomitant hyperlipidemia. Most studies reported the mean age of the patients, which mainly ranged from 35 to 72 years; the proportion of male patients was generally slightly higher. Regarding the intervention regimens, all studies used Xuesaitong injection combined with conventional biomedical therapy as the experimental intervention. The dose of Xuesaitong injection was most commonly 400 mg administered by intravenous infusion once daily, while a few studies used doses of 40 mL, 10 mL, 200 mg, or 500 mg. Available formulation-related information on Xuesaitong injection, including manufacturer, approval number, executive standard, main component, specification, and daily dose, was further extracted from the included studies and is summarized in Supplementary Material S3. The control groups received the same conventional treatment regimens as the experimental groups to ensure comparability between groups. The conventional therapeutic agents mainly included ulinastatin, octreotide acetate, fenofibrate, and somatostatin, whereas five studies did not clearly specify the drugs used. The treatment duration was mainly 7–10 days, with the longest duration being 14 days and the shortest being 5 days (Table 2).

**TABLE 2:** Characteristics of the included studies.

Study	Disease classification	Sample size (T/C)	Male proportion (T/C)	Mean age (T/C)	Mean disease duration (T/C), h	Xuesaitong regimen	Control regimen	Main outcomes	Treatment duration, days
Liu and Lu (2004)	Acute pancreatitis	32/32	10/12	35/36	NR	XST 400 mg iv.gtt qd	Conventional therapy	③	NR
Liu et al. (2006)	Acute pancreatitis	26/24	10/9	37/38	NR	XST 400 mg iv.gtt qd	Octreotide acetate	③⑨	7–10
Niu (2008)	Acute pancreatitis	37/32	NR	NR	NR	XST 500 mg iv.gtt qd	Conventional therapy	③	7–14
Du (2009)	Severe acute pancreatitis	50/51	29/28	57/60	NR	XST 400 mg iv.gtt qd	Conventional therapy	③	NR
Li et al. (2016)	Hyperlipidemic acute pancreatitis	30/30	20/21	35.2/36.9	NR	XST 400 mg iv.gtt qd	Conventional therapy	②③⑨	5
Fang et al. (2017)	Senile acute pancreatitis	40/40	22/23	67.25/68.06	5.12/5.84	XST 400 mg iv.gtt qd	Ulinastatin	①④⑥⑦⑧	14
Wang (2018)	Acute pancreatitis	53/53	36/34	52.35/53.19	5.26/5.31	XST 400 mg iv.gtt qd	Ulinastatin	①⑥⑧	14
Shi (2018)	Senile acute pancreatitis	30/30	16/12	72.3/72.1	6.1/5.1	XST 400 mg iv.gtt qd	Ulinastatin	①⑥⑧	14
Zhang (2018)	Senile acute pancreatitis	75/75	47/49	67.0/67.2	7.1/7.2	XST 40 mL iv.gtt qd	Ulinastatin	①②③⑤⑧	10
Zhang (2019)	Senile acute pancreatitis	48/48	29/28	68.43/68.41	5.11/5.07	XST 40 mL iv.gtt qd	Ulinastatin	①②④⑤⑦	10
Yu (2019)	Non-biliary mild acute pancreatitis	27/28	15/15	45.1/39.3	24.2/21.1	XST 40 mL iv.gtt qd	Somatostatin	①②⑤⑦⑨	7
Zhang (2020)	Hyperlipidemic acute pancreatitis	45/45	21/22	49/49	5.0/5.2	XST 400 mg iv.gtt qd	Fenofibrate	②④⑦	7
Lin (2020)	Senile acute pancreatitis	84/84	50/51	72.15/72.48	3.66/3.59	AP: XST 200 mg iv.gtt qd SAP: XST 400 mg iv.gtt qd	Ulinastatin	④⑦	14
Zou (2021)	Acute pancreatitis	120/120	74/78	46.39/45.76	NR	XST 400 mg iv.gtt qd	Octreotide acetate	①②③⑤	5
Song et al. (2021)	Acute pancreatitis	40/40	25/27	42.73/41.38	NR	XST 500 mg iv.gtt qd	Conventional therapy	①②④⑤⑦	7
Liu et al. (2024)	Acute pancreatitis	30/30	19/16	47.23/46.77	1.69/1.71	XST 400 mg iv.gtt qd	Somatostatin	②③④⑤⑦	7

NR, not reported; XST, Xuesaitong injection; iv.gtt, intravenous infusion; qd, once daily; ① TNF-α; ② CRP; ③ length of hospital stay; ④ serum amylase; ⑤ IL-6; ⑥ IL-2; ⑦ urinary amylase; ⑧ sICAM-1; ⑨ APACHE II.

### 3.3 Risk of bias

**FIGURE 2:**
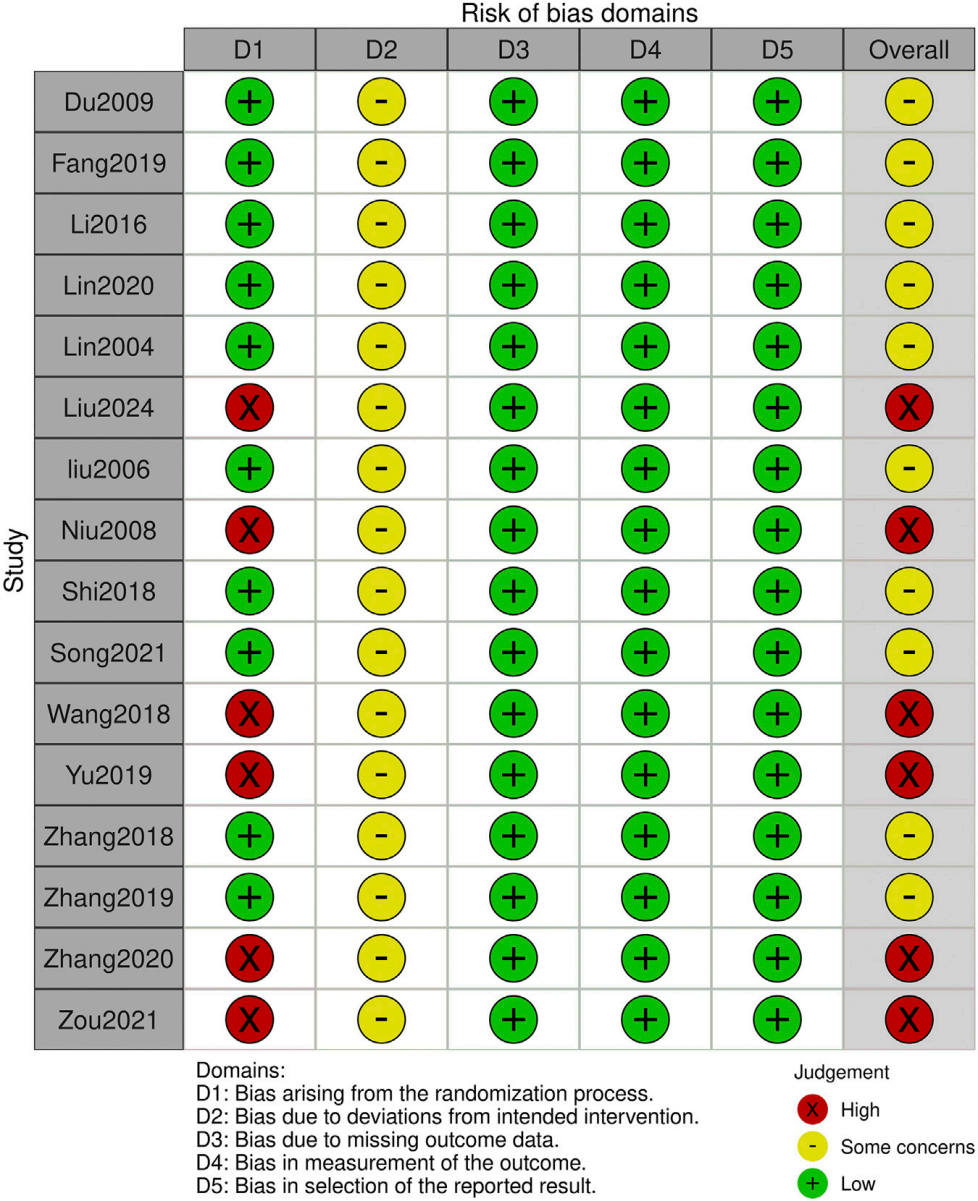
Results of the risk of bias assessment.

The RoB 2.0 assessment indicated that six of the 16 (Niu, 2008; Wang, 2018; Yu, 2019; Zhang, 2020; Zou, 2021; Liu et al., 2024) included trials were at high overall risk of bias, while the remaining 10 were judged as having some concerns; none was rated as low risk overall. The main methodological limitation was bias arising from the randomization process, with six studies judged as high risk in Domain 1 because of inadequate reporting or apparent problems in random sequence generation and/or allocation concealment. All studies were rated as having some concerns in Domain 2, reflecting insufficient information on deviations from intended interventions. By contrast, all included studies were judged to be at low risk in Domain 3 for missing outcome data, Domain 4 for outcome measurement, and Domain 5 for selective reporting. These findings suggest that the overall credibility of the pooled estimates may be mainly constrained by limitations in randomization and intervention-related methodological reporting (Figure 2).

### 3.4 Clinical response rate

**FIGURE 3:**
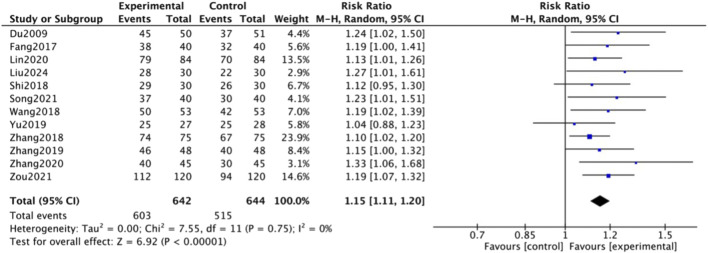
Clinical response rate.

Twelve studies (Du, 2009; Fang et al., 2017; Shi, 2018; Wang, 2018; Zhang, 2018; Zhang, 2019; Zhang, 2020; Yu, 2019; Lin, 2020; Song et al., 2021; Zou, 2021; Liu et al., 2024) reported the clinical response rate (n = 1,286). The clinical response rate was significantly higher in the intervention group than in the control group (RR = 1.15, 95% CI 1.11 to 1.20; *P* < 0.00001), with low heterogeneity (I^2^ = 0%) (Figure 3). Given that the criteria for determining clinical response rate differed across studies, the study-specific definitions were extracted and summarized in Supplementary Material S4.

### 3.5 Length of hospital stay

**FIGURE 4:**
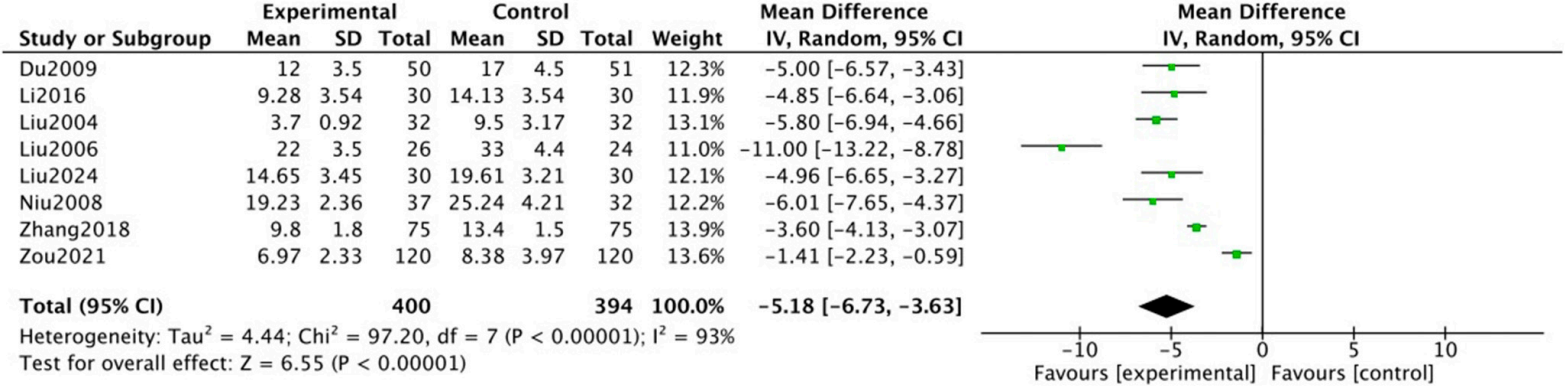
Length of hospital stay.

Eight studies (Liu and Lu, 2004; Liu et al., 2006; Liu et al., 2024; Niu, 2008; Du, 2009; Li et al., 2016; Zhang, 2018; Zou, 2021) reported the length of hospital stay (n = 794). The intervention group had a significantly shorter hospital stay than the control group (MD = −5.18 days, 95% CI −6.73 to −3.63; *P* < 0.00001), with substantial heterogeneity (I^2^ = 93%) (Figure 4).

### 3.6 Acute physiology and chronic health evaluation II (APACHE II) scores

**FIGURE 5:**
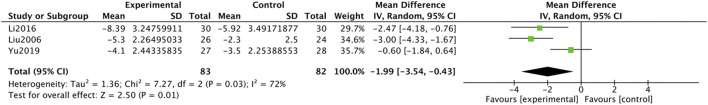
APACHE II scores.

Three studies (Liu et al., 2006; Li et al., 2016; Yu, 2019) reported APACHE II scores (n = 165). The intervention group had significantly lower APACHE II scores than the control group (MD = −1.99 points, 95% CI −3.54 to −0.43; *P* = 0.01), with substantial heterogeneity (I^2^ = 72%) (Figure 5).

### 3.7 Tumor necrosis factor-alpha (TNF-α)

**FIGURE 6:**
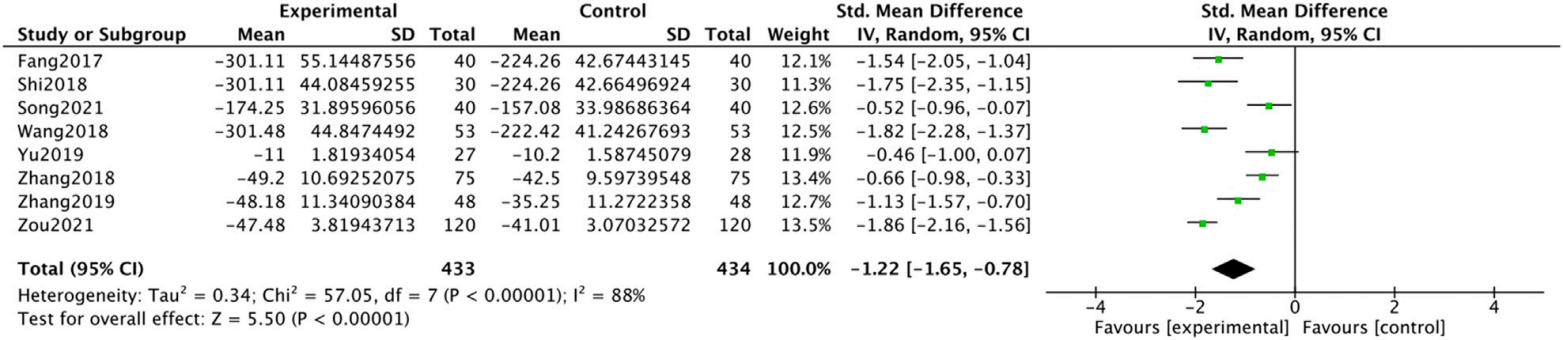
TNF-α.

Eight studies (Fang et al., 2017; Shi, 2018; Wang, 2018; Zhang, 2018; Zhang, 2019; Yu, 2019; Song et al., 2021; Zou, 2021) reported TNF-α levels (n = 867). TNF-α levels were significantly lower in the intervention group than in the control group (SMD = −1.22, 95% CI −1.65 to −0.78; *P* < 0.00001), with substantial heterogeneity (I^2^ = 88%) (Figure 6).

### 3.8 Inflammatory markers

**FIGURE 7:**
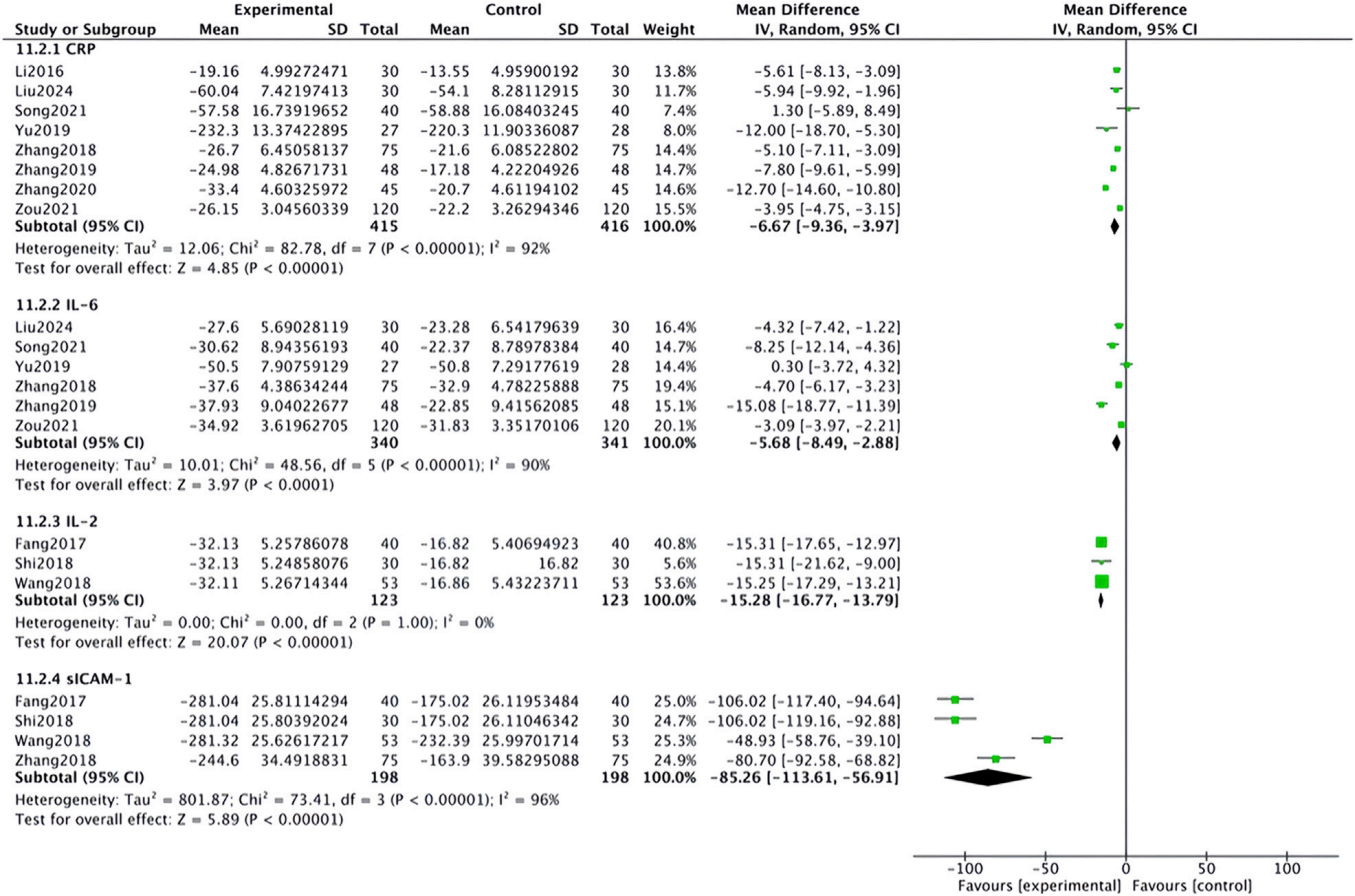
Inflammatory markers.

Eight studies (Li et al., 2016; Zhang, 2018; Zhang, 2019; Zhang, 2020; Yu, 2019; Song et al., 2021; Zou, 2021; Liu et al., 2024) reported C-reactive protein (CRP) levels (n = 831). CRP levels were significantly lower in the intervention group than in the control group (MD = −6.67 mg/L, 95% CI −9.36 to −3.97; *P* < 0.00001), with substantial heterogeneity (I^2^ = 92%). Six studies (Zhang, 2018; Zhang, 2019; Yu, 2019; Song et al., 2021; Zou, 2021; Liu et al., 2024) reported interleukin-6 (IL-6) levels (n = 681). IL-6 levels were significantly lower in the intervention group than in the control group (MD = −5.68 ng/L, 95% CI −8.49 to −2.88; *P* < 0.0001), with substantial heterogeneity (I^2^ = 90%). Three studies (Fang et al., 2017; Shi, 2018; Wang, 2018) reported interleukin-2 (IL-2) levels (n = 246). IL-2 levels were significantly lower in the intervention group than in the control group (MD = −15.28 pg/mL, 95% CI −16.77 to −13.79; *P* < 0.00001), with low heterogeneity (I^2^ = 0%). Four studies (Fang et al., 2017; Shi, 2018; Wang, 2018; Zhang, 2018) reported soluble intercellular adhesion molecule-1 (sICAM-1) levels (n = 396). sICAM-1 levels were significantly lower in the intervention group than in the control group (MD = −85.26 ng/mL, 95% CI −113.61 to −56.91; *P* < 0.00001), with substantial heterogeneity (I^2^ = 96%) (Figure 7).

### 3.9 Amylase

**FIGURE 8:**
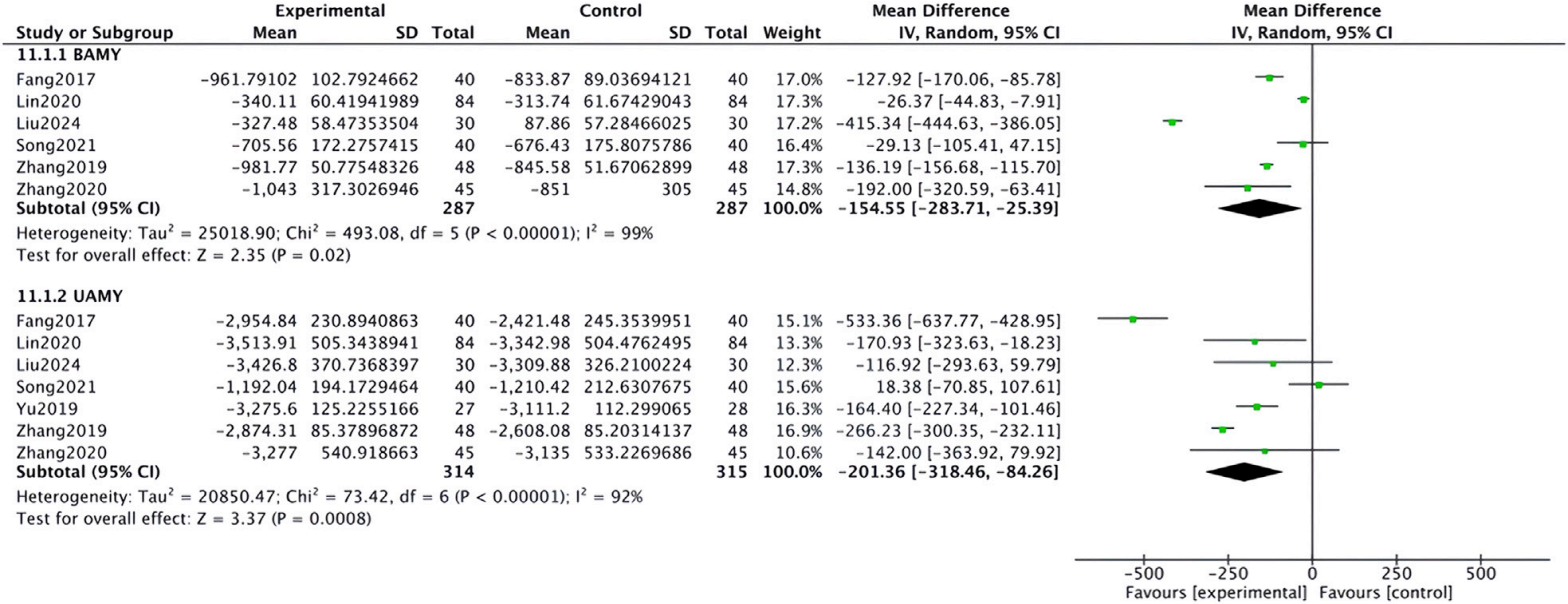
Amylase.

Six studies (Fang et al., 2017; Zhang, 2019; Zhang, 2020; Lin, 2020; Song et al., 2021; Liu et al., 2024) reported serum amylase levels (n = 574). Serum amylase levels were significantly lower in the intervention group than in the control group (MD = −154.55 U/L, 95% CI −283.71 to −25.39; *P* = 0.02), with substantial heterogeneity (I^2^ = 99%). Seven studies (Fang et al., 2017; Yu, 2019; Zhang, 2019; Zhang, 2020; Lin, 2020; Song et al., 2021; Liu et al., 2024) reported urinary amylase levels (n = 629). Urinary amylase levels were also significantly lower in the intervention group than in the control group (MD = −201.36 U/L, 95% CI −318.46 to −84.26; *P* = 0.0008), with substantial heterogeneity (I^2^ = 92%) (Figure 8).

### 3.10 Sensitivity analysis

After studies judged to have an overall high risk of bias were excluded and the effect estimates were recalculated, the direction of the pooled effects for all outcomes remained consistent with the original analyses, and no substantial changes in statistical significance were observed. Heterogeneity decreased for several outcomes, including TNF-α, CRP, and APACHE II score, after high-risk studies were excluded; in particular, the heterogeneity for APACHE II score decreased from 72% to 0%. In the leave-one-out analysis, exclusion of Liu 2006 (Liu et al., 2006) resulted in I^2^ = 67% and *P* = 0.12, whereas exclusion of Yu 2019 (Yu, 2019) resulted in I^2^ = 0% and *P* < 0.00001.

In addition, an exploratory sensitivity analysis was performed after excluding studies in which the background treatment clearly included ulinastatin, octreotide, somatostatin, or other non-pure supportive pharmacological interventions, retaining only studies in which the control regimen was conventional supportive care. For length of hospital stay, the direction of the pooled effect remained consistent with the original analysis and retained statistical significance, while heterogeneity decreased from 93% to 0% and *P* < 0.00001. For CRP, heterogeneity decreased from 92% to 68%, but the pooled effect was not statistically significant, with *P* = 0.37 (Table 3).

**TABLE 3:** Sensitivity analysis.

Outcome	Analysis	Patients (T/C)	RR/MD/SMD (95% CI)	Heterogeneity
Clinical response rate	Original analysis	642/644	1.15 [1.11, 1.20]	I^2^ = 0%, *P* < 0.00001
	Excluding studies with a high risk of bias	367/368	1.14 [1.08, 1.20]	I^2^ = 0%, *P* < 0.00001
Length of hospital stay	Original analysis	400/394	−5.18 [−6.73, −3.63]	I^2^ = 93%, *P* < 0.00001
	Excluding studies with a high risk of bias	213/212	−5.89 [−7.85, −3.92]	I^2^ = 92%, *P* < 0.00001
	Excluding studies with non-pure supportive care	149/145	−5.51 [−6.24, −4.77]	I^2^ = 0%, P < 0.00001
APACHE II	Original analysis	83/82	−1.99 [−3.54, −0.43]	I^2^ = 72%, *P* = 0.01
	Excluding studies with a high risk of bias	56/54	−2.80 [−3.85, −1.75]	I^2^ = 0%, *P* < 0.00001
	Exclusion of “Liu 2006”	57/58	−1.44 [−3.26, 0.38]	I^2^ = 67%, *P* = 0.12
	Exclusion of “Yu 2019”	56/54	−2.80 [−3.85, −1.75]	I^2^ = 0%, *P* < 0.00001
TNF-α	Original analysis	433/434	−1.22 [−1.65, −0.78]	I^2^ = 88%, *P* < 0.00001
	Excluding studies with a high risk of bias	233/233	−1.09 [−1.53, −0.64]	I^2^ = 79%, *P* < 0.00001
CRP	Original analysis	415/416	−6.67 [−9.36, −3.97]	I^2^ = 92%, *P* < 0.00001
	Excluding studies with a high risk of bias	193/193	−5.64 [−7.85, −3.43]	I^2^ = 65%, *P* < 0.00001
	Excluding studies with non-pure supportive care	70/70	−3.01 [−9.57, 3.55]	I^2^ = 68%, P = 0.37
IL-6	Original analysis	340/341	−5.68 [−8.49, −2.88]	I^2^ = 90%, *P* < 0.00001
	Excluding studies with a high risk of bias	163/163	−9.19 [−15.50, −2.88]	I^2^ = 93%, *P* = 0.004
IL-2	Original analysis	123/123	−15.28 [−16.77, −13.79]	I^2^ = 0%, *P* < 0.00001
	Excluding studies with a high risk of bias	70/70	−15.31 [−17.50, −13.12]	I^2^ = 0%, *P* < 0.00001
sICAM-1	Original analysis	198/198	−85.26 [−113.61, −56.91]	I^2^ = 96%, *P* < 0.00001
	Excluding studies with a high risk of bias	145/145	−97.52 [−114.25, −80.79]	I^2^ = 83%, *P* < 0.00001
Serum amylase	Original analysis	287/287	−154.55 [−283.71, −25.39]	I^2^ = 99%, *P* = 0.02
	Excluding studies with a high risk of bias	212/212	−82.07 [−152.32, −11.81]	I^2^ = 96%, *P* = 0.02
Urinary amylase	Original analysis	314/315	−201.36 [−318.46, −84.26]	I^2^ = 92%, *P* = 0.0008
	Excluding studies with a high risk of bias	212/212	−238.52 [−433.59, −43.44]	I^2^ = 95%, *P* = 0.02

### 3.11 Subgroup analysis

After subgroup analysis stratified by treatment duration, the direction of the pooled effects was generally consistent across most outcomes in the ≤7 days and >7 days subgroups, with both favoring the experimental group. Length of hospital stay, CRP, and urinary amylase all showed improvement trends in both subgroups, with the absolute effect size being larger in the >7 days subgroup. TNF-α and serum amylase reached statistical significance only in the >7 days subgroup, while IL-6 showed a significant difference only in the ≤7 days subgroup. The subgroup differences for all outcomes were not statistically significant. When stratified by mean age, the clinical response rate was significantly higher in both the ≥65 years and <65 years subgroups, with no heterogeneity observed in either subgroup. TNF-α and CRP were significantly reduced in both age subgroups. IL-6 was significantly reduced only in the <65 years subgroup, whereas serum amylase and urinary amylase were significantly reduced only in the ≥65 years subgroup. The between-subgroup difference by age was statistically significant only for urinary amylase, with *P* = 0.04, whereas no significant between-subgroup differences were observed for the other outcomes (Table 4).

**TABLE 4:** Subgroup analysis.

Outcome	Subgroup type	No. Of studies	Sample size	Effect size (95% CI)	*P*	I^2^	Interaction p value
Clinical response rate	Treatment duration ≤7 days	6	626	RR = 1.19 [1.11, 1.28]	<0.00001	0%	0.23
	Treatment duration >7 days	6	660	RR = 1.13 [1.08, 1.19]	<0.00001	0%	
	Age ≥65 years	5	554	RR = 1.13 [1.07, 1.19]	<0.00001	0	0.17
	Age <65 years	7	732	RR = 1.19 [1.12, 1.27]	<0.00001	0	
Length of hospital stay	Treatment duration ≤7 days	3	260	MD = −3.65 [−6.32, −0.98]	0.007	91%	0.20
	Treatment duration >7 days	3	269	MD = −6.75 [−10.63, −2.86]	0.0007	96%	
TNF-α	Treatment duration ≤7 days	3	275	SMD = −0.96 [−1.97, 0.04]	0.06	94%	0.49
	Treatment duration >7 days	5	292	SMD = −1.36 [−1.84, −0.88]	<0.00001	82%	
	Age ≥65 years	4	286	SMD = −1.23 [−1.73, −0.74]	<0.00001	79	0.91
	Age <65 years	4	481	SMD = −1.18 [−1.94, −0.42]	0.002	92	
CRP	Treatment duration ≤7 days	6	585	MD = −6.68 [−10.70, −2.66]	0.001	93%	0.46
	Treatment duration >7 days	2	151	MD = −8.61 [−11.86, −5.36]	<0.00001	29%	
	Age ≥65 years	2	246	MD = −6.49 [−9.13, −3.84]	<0.00001	74	0.94
	Age <65 years	6	585	MD = −6.68 [−10.70, −2.66]	0.001	93	
IL-6	Treatment duration ≤7 days	4	435	MD = −0.65 [−1.04, −0.25]	0.001	70%	0.79
	Treatment duration >7 days	2	246	MD = −0.30 [−2.88, 2.29]	0.82	99%	
	Age ≥65 years	2	246	MD = −9.75 [−19.91, 0.42]	0.06	96	0.26
	Age <65 years	4	535	MD = −3.76 [−6.29, −1.24]	0.003	70	
Serum amylase	Treatment duration ≤7 days	3	230	MD = −213.96 [−492.39, 64.48]	0.13	98%	0.43
	Treatment duration >7 days	3	242	MD = −96.04 [−177.17, −14.90]	0.02	97%	
	Age ≥65 years	4	434	MD = −111.08 [−185.72, −36.45]	0.004	96	0.57
	Age <65 years	2	140	MD = −223.91 [−602.37,154.56]	0.25	99	
Urinary amylase	Treatment duration ≤7 days	4	285	MD = −137.82 [−266.48, −9.16]	0.04	92%	0.10
	Treatment duration >7 days	3	344	MD = −326.9 [−515.51, −138.17]	0.0007	92%	
	Age ≥65 years	3	344	MD = −326.90 [−515.51, −138.28]	0.0007	92	0.04
	Age <65 years	4	285	MD = −95.26 [−207.07, 16.56]	0.09	72	

### 3.12 Trial sequential analysis

Trial sequential analysis was performed for clinical response rate, length of hospital stay, serum amylase (BAMY), urinary amylase (UAMY), CRP, IL-6, sICAM-1, and TNF-α. For clinical response rate, length of hospital stay, UAMY, CRP, IL-6, sICAM-1, and TNF-α, the cumulative Z-curves crossed the conventional significance boundary and reached or crossed the trial sequential monitoring boundary under the prespecified assumptions, supporting the robustness of the pooled findings and suggesting that the risk of random error was limited. For BAMY, the cumulative Z-curve crossed the conventional significance boundary but did not clearly cross the trial sequential monitoring boundary, indicating that although the pooled effect was statistically significant, the evidence may still be insufficient to confirm a firm conclusion after adjustment for random error. For IL-2, the official TSA software was unable to render the graph because the first information fraction exceeded 100% of the required information size. This indicates that the accrued information at the first cumulative analysis was already larger than the estimated required information size, making the monitoring boundary non-renderable in the software. Therefore, IL-2 was not included in the combined TSA figure; this was a software-rendering limitation under the selected assumptions rather than evidence that the original meta-analysis result was invalid (Supplementary Image 1).

### 3.13 Safety

Eight studies (Liu et al., 2006; Liu et al., 2024; Li et al., 2016; Fang et al., 2017; Shi, 2018; Zhang, 2018; Yu, 2019; Lin, 2020) reported safety outcomes. The reported adverse drug reactions mainly included allergic reactions, rash, leukopenia, abnormal liver function, nausea, and chest distress. In addition, one study reported three cases of conversion to surgery in each group and one death in the control group, while another study reported three cases of pseudocyst formation in the experimental group. Overall, adverse events occurred in both groups and were mostly mild to moderate. The overall incidence of reported safety-related events was 6.43% in the experimental group and 7.04% in the control group. When only adverse drug reactions were considered, most reported events were mild to moderate. No serious adverse events clearly attributed to Xuesaitong injection were reported in the included studies (Table 5).

**TABLE 5:** Safety-related outcomes reported in the included studies.

Study	Sample size (T/C)	Experimental group	Control group	Incidence (T/C)
Liu et al. (2006)	26/24	Converted to surgery (3 cases)	Converted to surgery (3 cases), death (1 case)	11.54%/16.67%
Li et al. (2016)	30/30	Leukopenia (2 cases)	Rash (1 case)	6.67%/3.33%
Fang et al. (2017)	40/40	Rash (1 case), leukopenia (1 case)	Rash (2 cases), leukopenia (1 case)	5.00%/7.50%
Shi (2018)	30/30	Rash (1 case), leukopenia (1 case)	Rash (2 cases), leukopenia (5 cases)	6.67%/23.33%
Zhang (2018)	75/75	Rash (1 case), abnormal liver function (2 cases)	Rash (1 case)	4.00%/1.33%
Yu (2019)	27/28	Leukopenia (2 cases), rash (1 case)	Rash (1 case), abnormal liver function (1 case)	11.11%/7.14%
Lin (2020)	84/84	Rash (3 cases), nausea (1 case)	Rash (2 cases), leukopenia (1 case)	4.76%/3.57%
Liu et al. (2024)	30/30	Rash (2 cases), chest discomfort (1 case)	Rash (2 cases), nausea (1 case)	10.00%/10.00%
Total	342/341	22 cases	24 cases	6.43%/7.04%

### 3.14 Publication bias

**FIGURE 9:**
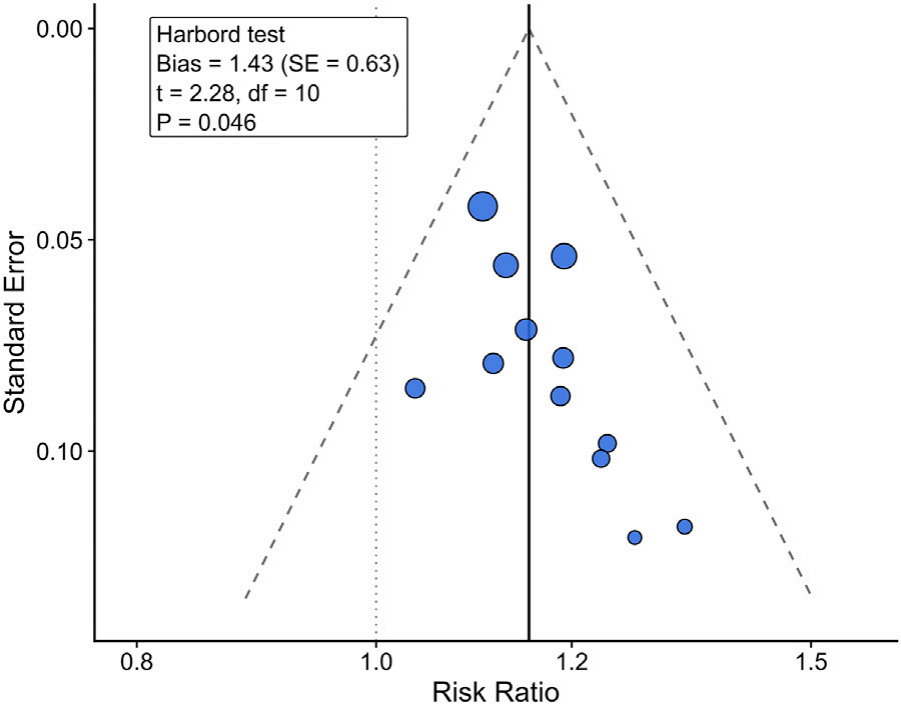
Funnel plot for the clinical response rate.

Publication bias was assessed among the 12 studies that reported the clinical response rate. The funnel plot showed some asymmetry in the distribution of effect estimates. Harbord’s regression test for binary outcomes was further performed to assess small-study effects, showing Bias = 1.43, SE = 0.63, t = 2.28, df = 10, and *P* = 0.046. These findings suggest possible small-study effects or publication bias for this outcome (Figure 9).

### 3.15 Quality of evidence

The Grading of Recommendations Assessment, Development and Evaluation (GRADE) results indicated that the quality of evidence for the clinical response rate was low, suggesting that Xuesaitong injection may offer some benefit in improving the overall clinical response in patients with acute pancreatitis. The quality of evidence for the remaining outcomes, including adverse events, length of hospital stay, APACHE II score, TNF-α, CRP, IL-6, IL-2, sICAM-1, serum amylase, and urinary amylase, was very low. This indicates that the current evidence on the effect of Xuesaitong injection in improving disease severity, inflammatory responses, endothelial injury, and pancreatic enzyme-related markers is limited, and the results should be interpreted with caution. For further details, please refer to the Supplementary Material S2.

## 4 Discussion

The results of this meta-analysis indicate that the addition of Xuesaitong injection to conventional treatment for acute pancreatitis may improve clinical response rates, shorten hospital stay, reduce APACHE II scores, and improve pancreatic enzyme and inflammation-related markers. These findings suggest that Xuesaitong injection, as an adjunctive therapy for acute pancreatitis, may have potential advantages in alleviating inflammation and mitigating disease severity. However, the criteria used to define clinical response rate varied across studies, and this variability may reduce the interpretability and certainty of this outcome.

The pathological progression of acute pancreatitis is considered a complex process that evolves from local injury to a systemic inflammatory response, rather than being confined solely to the pancreas. After injury, acinar cells may undergo abnormal activation of zymogens and cell death due to calcium homeostasis imbalance and mitochondrial dysfunction, leading to the release of damage-associated molecules. This results in abnormal release of pancreatic enzymes, accompanied by elevated serum and urinary amylase levels (Zhu et al., 2024; Srinivasamurthy et al., 2026). This triggers the body’s inflammatory response, activating the innate immune system, and amplifies the inflammatory cascade through signaling pathways such as NF-κB. This promotes the release of inflammatory cytokines like TNF-α and IL-6, as well as the elevation of inflammation- and endothelial injury-related markers such as CRP and sICAM-1 (Liu T. et al., 2023; Papantoniou et al., 2024; Hu et al., 2026). As the inflammatory response progresses, endothelial function and the microcirculatory system are gradually impaired, manifested by increased capillary permeability, leukocyte adhesion, and microthrombus formation. This ultimately exacerbates local pancreatic ischemia and damage to other organs (Hu et al., 2026).

The main active constituents of Xuesaitong injection are *Panax notoginseng* saponins. Existing studies have shown that it has multi-target pharmacological effects, primarily exerting its actions by inhibiting inflammatory responses, improving microcirculation, and protecting endothelial function (He et al., 2022; Li S. et al., 2023). In addition, some studies suggest that it may modulate immune responses, playing a role in the regulation of the inflammatory process. In experimental models of acute pancreatitis, *Panax notoginseng* saponins can alleviate inflammation in the pancreas and multiple organs by inhibiting the activation of signaling pathways such as NF-κB and downregulating the expression of pro-inflammatory cytokines like TNF-α and IL-6 (Liu et al., 2018). In the pathological progression, microcirculatory dysfunction and endothelial injury are considered key factors driving organ failure (Hu et al., 2026). *Panax notoginseng* saponins may improve microcirculation and reduce pancreatic and other organ damage by inhibiting oxidative stress, reducing the adhesion of leukocytes to endothelial cells, downregulating the abnormal expression of sICAM-1, and promoting blood flow (Zhao et al., 2021; He et al., 2022; Liu P. et al., 2023), thereby alleviating organ injury (Hu et al., 2026). *Panax notoginseng* saponins may, to some extent, reduce acinar cell necrosis and the leakage of pancreatic enzymes by improving pancreatic microcirculatory perfusion, alleviating the inflammatory cascade, and reducing oxidative stress damage. This, in turn, may indirectly impact the abnormal elevation of serum and urinary amylase levels (Liu et al., 2018). In addition, it may also exert effects by modulating immune responses, such as regulating the transformation of microglia from the M1 to the M2 phenotype through the phosphoinositide 3-kinase/protein kinase B (PI3K/Akt) signaling pathway, inhibiting the activation of pro-inflammatory macrophages, thereby alleviating ischemia-reperfusion injury and mitigating both local and systemic inflammatory responses induced by acute pancreatitis (Li et al., 2019; Li et al., 2023a). From an ethnopharmacological perspective, the potential rationale for using *Panax notoginseng* in acute pancreatitis may be partly explained by its traditional therapeutic characteristics. Traditionally, *Panax notoginseng* has been used to improve impaired blood flow, reduce swelling, and relieve pain. These traditional uses are partially consistent with the clinical and pathological features of acute pancreatitis, which commonly include marked abdominal pain, local tissue edema, microcirculatory dysfunction, and inflammation-related organ injury. Taken together with existing basic research indicating that *Panax notoginseng* saponins may modulate pancreatic inflammatory responses and pro-inflammatory cytokines, the use of Xuesaitong injection and *Panax notoginseng* saponins as adjuvant therapy for acute pancreatitis appears to have a certain traditional therapeutic basis and modern pharmacological rationale (Mancuso, 2024; Kim et al., 2025).

When subgroup analyses were performed according to treatment duration of ≤7 days or >7 days, no statistically significant between-subgroup differences were observed for any outcome, suggesting that treatment duration may not be a major contributor to heterogeneity. When subgroup analyses were performed according to mean age, most outcomes showed no statistically significant between-subgroup differences, suggesting that age stratification may not sufficiently explain the heterogeneity observed across most outcomes. Although urinary amylase showed a statistically significant between-subgroup difference by age, with a larger absolute effect size in the ≥65 years subgroup, this result should be interpreted cautiously. The number of studies included after stratification was limited, and substantial heterogeneity remained within each subgroup; therefore, it cannot be concluded that elderly patients are more likely to benefit. In addition, differences in the dosage and formulation of Xuesaitong injection may also have contributed to heterogeneity. We further extracted the available information on manufacturer, approval number, executive standard, main component, specification, and daily dose from the included studies, and found variations across studies in manufacturer, specification, and dose-reporting format. Although most studies reported *Panax notoginseng* saponins as the main component, the dosage was reported inconsistently across studies, with some using mg/day and others using mL/day. In particular, (Zhang 2019; Yu, 2019) reported only 40 mL/day without specifying the concentration or formulation specification; therefore, these doses could not be reliably converted into mg-based doses. Therefore, dose-based subgroup analysis and meta-regression were not performed. We also assessed the feasibility of exploring heterogeneity according to AP subtype and disease severity. However, because the number of studies within each subtype was limited and AP subtype overlapped with factors such as age, background treatment regimen, dosage, and treatment duration, a formal AP subtype-based subgroup analysis was not conducted. Future studies should prespecify subgroups according to age, disease severity, etiological classification, and dosage at the design stage, and should standardize the reporting of Xuesaitong injection-related information, so as to further clarify the patient characteristics most likely to benefit and the optimal dosing and treatment regimen.

The sensitivity analysis in this study showed that for most outcomes, the direction of the pooled effects and the level of heterogeneity remained largely unchanged after using the leave-one-out method, excluding high-risk studies, and switching the effect model. For the APACHE II score, after excluding “Yu 2019 (Yu, 2019)”, the I^2^ decreased from 72% to 0%, suggesting that this study may be the primary source of heterogeneity. After excluding “Liu 2006 (Liu et al., 2006)”, the pooled effect lost statistical significance, indicating that this study had a significant impact on the overall effect. A combined analysis of the original data from three studies indicated that “Yu 2019 (Yu, 2019)” included patients with mild non-biliary acute pancreatitis, who had relatively mild conditions and low baseline scores. After basic treatment, the APACHE II scores had already significantly improved, and the incremental effect of adding Xuesaitong injection was limited. In contrast, “Liu 2006 (Liu et al., 2006)” and “Li 2016 (Li et al., 2016)” included patients with severe pancreatitis and hyperlipidemic acute pancreatitis, respectively, who had higher baseline scores and more severe conditions. The effect of Xuesaitong adjuvant therapy was more pronounced in these patients. In addition, the three studies also differed in terms of the control regimens, the dosage of Xuesaitong used, and the time points for outcome evaluation, which may have contributed to the observed heterogeneity. In addition, because some included studies used ulinastatin, octreotide, or somatostatin as part of the background treatment, all of which have anti-inflammatory or antitrypsin activity, an exploratory sensitivity analysis was performed by retaining only studies in which the control regimen consisted of conventional supportive care. For length of hospital stay, the direction of the pooled effect remained consistent with the original analysis and retained statistical significance, with heterogeneity decreasing from 93% to 0%. This suggests that differences in background treatment regimens may have been an important source of heterogeneity for this outcome. In contrast, although heterogeneity for CRP decreased after excluding the relevant studies, the pooled effect was no longer statistically significant. This finding suggests that inflammation-related outcomes may be more sensitive to differences in treatment regimens, patient subtypes, and the reduced number of remaining studies. Therefore, conclusions regarding the improvement of inflammatory markers should be interpreted with caution.

In addition, trial sequential analysis was performed to evaluate the robustness of the cumulative evidence and the risk of random errors. The cumulative Z-curves for clinical response rate, length of hospital stay, urinary amylase, CRP, IL-6, sICAM-1, and TNF-α crossed the conventional significance boundary and reached or crossed the trial sequential monitoring boundary, suggesting that, under the prespecified assumptions, the risk of random error for these outcomes may be relatively low. For serum amylase, the cumulative Z-curve crossed the conventional significance boundary but did not clearly cross the trial sequential monitoring boundary, indicating that the evidence for this outcome may still be insufficient to support a firm conclusion after adjustment for random error. However, TSA primarily addresses random error and cannot overcome limitations related to risk of bias, clinical heterogeneity, publication bias, and low certainty of evidence in the included studies. Therefore, these findings should still be interpreted cautiously together with the GRADE assessment. For IL-2, the trial sequential monitoring graph could not be generated by the software because the first information fraction had already exceeded the required information size. Therefore, this outcome was not included in the combined TSA figure.

In the studies included in this meta-analysis, the reported safety-related outcomes mainly included skin reactions, leukopenia, abnormal liver function, pseudocyst formation, nausea, chest distress, as well as clinical safety-related outcomes such as conversion to surgery and death. Among these events, rash, leukopenia, abnormal liver function, nausea, and chest distress were more likely to represent adverse drug reactions. In contrast, conversion to surgery and death reported in Liu 2006 (Liu et al., 2006) should be considered safety-related clinical outcomes, and the original study did not clearly attribute these events to Xuesaitong injection. Overall, among the included RCTs, the incidence of reported safety-related events was 6.43% in the experimental group and 7.04% in the control group. When only adverse drug reactions were considered, most reported events appeared to be mild to moderate.

In addition, previous studies have suggested that the overall incidence of adverse reactions to Xuesaitong injection is approximately 0.33%, with most reactions occurring in the skin and appendage systems, such as rashes and erythema (He et al., 2020). However, Dou 2025 and Wu 2022 both indicated that anaphylactic shock may occur in association with Xuesaitong injection (Wu X. -W. et al., 2022; Dou et al., 2025). From the perspective of potential mechanisms, Hao 2022 suggested that the allergenic potential of Xuesaitong injection may be related to certain rare saponins, such as gypenoside LXXV, notoginsenoside T5, notoginsenoside ST-4, and ginsenoside Rk3, which may contribute to anaphylactoid reactions by directly stimulating effector cell degranulation. In addition, severe adverse reactions associated with Xuesaitong injection are unlikely to be attributable to a single component alone, but may instead involve multiple factors, including rare saponins, formulation quality, excipients or impurities, administration dose and infusion rate, concomitant medications, and individual allergic susceptibility (Hao et al., 2022). Although the adverse events reported in the RCTs included in this meta-analysis were generally infrequent and mostly mild to moderate, the limited sample sizes of the original studies and insufficient adverse event reporting mean that a definitive conclusion on the safety of Xuesaitong injection in patients with acute pancreatitis cannot be drawn. Therefore, in clinical practice, it is essential to strictly follow the indications, adhere to standardized dosing protocols, and strengthen monitoring of high-risk patients to reduce the risk of adverse reactions.

This study still has some limitations. First, the overall methodological quality of the included studies was limited, with six studies being rated as having a high risk of bias, primarily due to insufficient reporting of information regarding the randomization process, allocation concealment, and intervention implementation. In addition, all 16 included studies were conducted in mainland China, and most were small, single-center trials with generally positive findings. Publication bias could be assessed only for the clinical response rate, for which funnel plot asymmetry and Harbord’s test suggested possible small-study effects or publication bias. For the remaining outcomes, the limited number of included studies precluded reliable assessment of publication bias, which was one of the reasons for downgrading the certainty of evidence in the GRADE assessment. Moreover, because all included studies were conducted in mainland China, the generalizability of the findings to other countries, regions, and racial or ethnic populations remains uncertain and requires further validation. Second, several continuous outcomes exhibited varying degrees of statistical heterogeneity. Although sensitivity analyses suggested that the direction of the overall effect was stable, outcomes such as length of hospital stay, serum amylase, urinary amylase, and IL-6 still showed significant heterogeneity after excluding high-risk studies, indicating that the results may have been influenced by a combination of clinical and methodological differences. From a clinical perspective, the characteristics of the populations included in the studies were not entirely consistent, as they involved patients with acute pancreatitis of varying etiologies and severities. Some studies included patients with hyperlipidemic or non-biliary acute pancreatitis. Additionally, there were variations in the dosage, treatment duration, and combination therapy regimens for Xuesaitong injection, and differences in the control measures may have also affected the consistency of the pooled effects. From a methodological perspective, differences across studies in the time points for outcome measurement, laboratory testing methods, and criteria for determining clinical response rate may have further contributed to the statistical heterogeneity. Third, the limited number of studies and sample sizes for some key outcomes affected the precision of the result estimates. Fourth, among the randomized controlled trials included in this study, only one study reported mortality and conversion to surgery, whereas the remaining studies did not report clinically important severity-related outcomes, such as mortality, persistent organ failure, infected pancreatic necrosis, or ICU admission. This limits the completeness of the clinical evidence to some extent. Future studies should include not only conventional efficacy indicators but also severity-related clinical outcomes, to allow a more comprehensive evaluation of the efficacy and safety of Xuesaitong injection as an adjuvant therapy for acute pancreatitis.

## 5 Conclusion

Xuesaitong injection, as an adjunctive therapy for acute pancreatitis, may improve clinical response rates and offer benefits in shortening hospital stay, reducing APACHE II scores, and improving inflammation- and enzyme-related markers. However, the certainty of the current evidence remains limited because of the risk of bias in the included studies, substantial heterogeneity, potential publication bias, and insufficient reporting of adverse events. In addition, the specific patient subtypes most likely to benefit, as well as the optimal dose and treatment duration, remain unclear. Therefore, multicenter, large-sample, double-blind randomized controlled trials are needed to further validate its efficacy and safety.

## Data Availability

The raw data supporting the conclusions of this article will be made available by the authors, without undue reservation.
