# Supplementary material for: Comparative efficacy and safety of Xuesaitong injection as adjuvant therapy for acute pancreatitis: a systematic review and meta-analysis
Source: Front Pharmacol. 2026 Jul 29;17:1875983. doi: 10.3389/fphar.2026.1875983 (PMC13462745; doi:10.3389/fphar.2026.1875983)
Supplement: Supplementary file 1 [file Table1.docx]

**Supplementary File 1. Search strategies for each database**

| **Database** | **Search field** | **Search strategy** |
| --- | --- | --- |
| PubMed | Title/Abstract; MeSH terms where applicable | (("Xuesaitong"[Title/Abstract] OR "Xue Sai Tong"[Title/Abstract] OR "Xuesaitong injection"[Title/Abstract] OR "Xuesaitong Injection"[Title/Abstract])  AND  ("Pancreatitis"[Mesh] OR "acute pancreatitis"[Title/Abstract] OR pancreatitis[Title/Abstract] OR "severe acute pancreatitis"[Title/Abstract])) |
| Embase | Title/Abstract/Keyword; Emtree terms where applicable | (('xuesaitong':ti,ab,kw OR 'xue sai tong':ti,ab,kw OR 'xuesaitong injection':ti,ab,kw)  AND  ('pancreatitis'/exp OR 'acute pancreatitis':ti,ab,kw OR pancreatitis:ti,ab,kw OR 'severe acute pancreatitis':ti,ab,kw)) |
| Cochrane Library | Title/Abstract/Keyword | ((Xuesaitong OR "Xue Sai Tong" OR "Xuesaitong injection")  AND  ("acute pancreatitis" OR pancreatitis OR "severe acute pancreatitis")) |
| Web of Science | Topic | TS=("Xuesaitong" OR "Xue Sai Tong" OR "Xuesaitong injection" OR "Xuesaitong Injection")  AND  TS=("acute pancreatitis" OR pancreatitis OR "severe acute pancreatitis") |
| China National Knowledge Infrastructure (CNKI) | Subject/Topic | SU=(血塞通 OR 血塞通注射液 OR 注射用血塞通 OR 血塞通冻干粉针 OR 注射用血塞通冻干)  AND  SU=(急性胰腺炎 OR 重症急性胰腺炎 OR 重症胰腺炎 OR 高脂血症性急性胰腺炎 OR 非胆源性急性胰腺炎) |
| Wanfang Data | Subject | (主题:血塞通 OR 主题:血塞通注射液 OR 主题:注射用血塞通 OR 主题:血塞通冻干粉针 OR 主题:注射用血塞通冻干)  AND  (主题:急性胰腺炎 OR 主题:重症急性胰腺炎 OR 主题:重症胰腺炎 OR 主题:高脂血症性急性胰腺炎 OR 主题:非胆源性急性胰腺炎) |
| VIP Database | Title/Keyword/Abstract | (M=血塞通 OR M=血塞通注射液 OR M=注射用血塞通 OR M=血塞通冻干粉针 OR M=注射用血塞通冻干)  AND  (M=急性胰腺炎 OR M=重症急性胰腺炎 OR M=重症胰腺炎 OR M=高脂血症性急性胰腺炎 OR M=非胆源性急性胰腺炎) |
| Chinese Biomedical Literature Database (CBM) | Common fields | ((血塞通[常用字段] OR 血塞通注射液[常用字段] OR 注射用血塞通[常用字段] OR 血塞通冻干粉针[常用字段] OR 注射用血塞通冻干[常用字段])  AND  (急性胰腺炎[常用字段] OR 重症急性胰腺炎[常用字段] OR 重症胰腺炎[常用字段] OR 高脂血症性急性胰腺炎[常用字段] OR 非胆源性急性胰腺炎[常用字段])) |

Note: XST, Xuesaitong injection; MeSH, Medical Subject Headings; Emtree, Embase subject headings; CNKI, China National Knowledge Infrastructure; VIP, Chinese Science and Technology Journal Database; CBM, Chinese Biomedical Literature Database. Search syntaxes may require minor field-tag adaptation according to the interface version of each database.
