# Supplementary material for: Comparative efficacy and safety of Xuesaitong injection as adjuvant therapy for acute pancreatitis: a systematic review and meta-analysis
Source: Front Pharmacol. 2026 Jul 29;17:1875983. doi: 10.3389/fphar.2026.1875983 (PMC13462745; doi:10.3389/fphar.2026.1875983)
Supplement: Supplementary file 2 [file Table2.docx]

**Supplementary File2. GRADE Summary of Findings and Evidence Profile**

**1. Summary of findings**

| **Outcome** | **Risk with control** | **Risk / mean difference with intervention** | **Relative or absolute effect (95% CI)** | **Participants (studies)** | **Certainty of the evidence (GRADE)** | **Comments** |
| --- | --- | --- | --- | --- | --- | --- |
| Clinical response rate | 800 per 1,000 | 920 per 1,000 (888 to 960) | RR 1.15 (1.11 to 1.20) | 1,286 (12 RCTs) | LOW ⊕⊕◯◯ | Adjunctive Xuesaitong may improve the overall clinical response. |
| Adverse events | Not estimable | Not estimable | Not pooled | Not reliably estimable (8 RCTs reported AEs) | VERY LOW ⊕◯◯◯ | Adverse events were generally mild to moderate, but definitions, ascertainment, and reporting were inconsistent, preventing a reliable pooled estimate. |
| Length of hospital stay | — | Mean 5.18 days lower (6.73 lower to 3.63 lower) | MD -5.18 days (-6.73 to -3.63) | 794 (8 RCTs) | VERY LOW ⊕◯◯◯ | Hospital stay appeared shorter with adjunctive treatment, but confidence in the estimate is markedly limited by heterogeneity. |
| APACHE II score | — | Mean 1.99 points lower (3.54 lower to 0.43 lower) | MD -1.99  (-3.54 to -0.43) | 165 (3 RCTs) | VERY LOW ⊕◯◯◯ | Disease severity score may decrease, but the evidence base is small and inconsistent. |
| TNF-α | — | Mean 1.22 SD lower (1.65 lower to 0.78 lower) | SMD -1.22 (-1.65 to -0.78) | 867 (8 RCTs) | VERY LOW ⊕◯◯◯ | TNF-α may decrease, although this is a surrogate biomarker and the estimate was highly heterogeneous. |
| CRP | — | Mean 6.67 units lower (9.36 lower to 3.97 lower) | MD -6.67 (-9.36 to -3.97) | 831 (8 RCTs) | VERY LOW ⊕◯◯◯ | CRP may decrease, but confidence is very limited because of heterogeneity and study limitations. |
| IL-6 | — | Mean 5.68 units lower (8.49 lower to 2.88 lower) | MD -5.68 (-8.49 to -2.88) | 681 (6 RCTs) | VERY LOW ⊕◯◯◯ | IL-6 may decrease; however, certainty is very low. |
| IL-2 | — | Mean 15.28 units lower (16.77 lower to 13.79 lower) | MD -15.28 (-16.77 to -13.79) | 246 (3 RCTs) | VERY LOW ⊕◯◯◯ | IL-2 may decrease, but this evidence comes from only three small trials and reflects a surrogate inflammatory endpoint. |
| Serum amylase (BAMY) | — | Mean 154.55 units lower (283.71 lower to 25.39 lower) | MD -154.55 (-283.71 to -25.39) | 574 (6 RCTs) | VERY LOW ⊕◯◯◯ | Blood amylase may decrease, but the estimate is highly unstable across studies. |
| Urine amylase (UAMY) | — | Mean 201.36 units lower (318.46 lower to 84.26 lower) | MD -201.36 (-318.46 to -84.26) | 629 (7 RCTs) | VERY LOW ⊕◯◯◯ | Urine amylase may decrease, but the certainty of evidence is very low. |
| sICAM-1 | — | Mean 85.26 units lower (113.61 lower to 56.91 lower) | MD -85.26 (-113.61 to -56.91) | 396 (4 RCTs) | VERY LOW  ⊕◯◯◯ | sICAM-1 may decrease, although this is a surrogate biomarker of endothelial and inflammatory injury and the estimate was highly heterogeneous. |

Notes: CI, confidence interval; MD, mean difference; RR, risk ratio; SD, standard deviation; SMD, standardized mean difference; RCT, randomized controlled trial. The control risk for clinical response rate was calculated from the pooled event rate in the control arm (515/644). Continuous outcomes are expressed as mean differences or standardized mean differences because a control-group baseline mean was not consistently available for all studies. Adverse events were not meta-analyzed because the original reports did not consistently provide participant-level total event counts, severity grading, or harmonized definitions.

**2. GRADE evidence profile**

| **Outcome** | **Study design** | **Risk of bias** | **Inconsistency** | **Indirectness** | **Imprecision** | **Other considerations** | **Participants (studies)** | **Effect (95% CI)** | **Certainty** |
| --- | --- | --- | --- | --- | --- | --- | --- | --- | --- |
| Clinical response rate | RCTs | Serious | Not serious | Not serious | Not serious | Publication bias strongly suspected | 1,286 (12) | RR 1.15 (1.11 to 1.20) | LOW ⊕⊕◯◯ |
| Adverse events | RCTs | Serious | Serious | Not serious | Very serious | Publication bias strongly suspected | Not reliably estimable (8) | Not pooled | VERY LOW ⊕◯◯◯ |
| Length of hospital stay | RCTs | Serious | Very serious | Not serious | Not serious | Publication bias strongly suspected | 794 (8) | MD -5.18 (-6.73 to -3.63) | VERY LOW ⊕◯◯◯ |
| APACHE II score | RCTs | Serious | Serious | Not serious | Serious | Publication bias strongly suspected | 165 (3) | MD -1.99 (-3.54 to -0.43) | VERY LOW ⊕◯◯◯ |
| TNF-α | RCTs | Serious | Very serious | Serious | Not serious | Publication bias strongly suspected | 867 (8) | SMD -1.22 (-1.65 to -0.78) | VERY LOW ⊕◯◯◯ |
| CRP | RCTs | Serious | Very serious | Serious | Not serious | Publication bias strongly suspected | 831 (8) | MD -6.67 (-9.36 to -3.97) | VERY LOW ⊕◯◯◯ |
| IL-6 | RCTs | Serious | Very serious | Serious | Not serious | Publication bias strongly suspected | 681 (6) | MD -5.68 (-8.49 to -2.88) | VERY LOW ⊕◯◯◯ |
| IL-2 | RCTs | Serious | Not serious | Serious | Serious | Publication bias strongly suspected | 246 (3) | MD -15.28 (-16.77 to -13.79) | VERY LOW ⊕◯◯◯ |
| Blood amylase (BAMY) | RCTs | Serious | Very serious | Serious | Not serious | Publication bias strongly suspected | 574 (6) | MD -154.55 (-283.71 to -25.39) | VERY LOW ⊕◯◯◯ |
| Urine amylase (UAMY) | RCTs | Serious | Very serious | Serious | Not serious | Publication bias strongly suspected | 629 (7) | MD -201.36 (-318.46 to -84.26) | VERY LOW ⊕◯◯◯ |
| sICAM-1 | RCTs | Serious | Very serious | Serious | Not serious | Publication bias strongly suspected | 396 (4) | MD -85.26 (-113.61 to -56.91) | VERY LOW ⊕◯◯◯ |

*Reasons for downgrading: serious risk of bias because none of the included trials was judged to be at low overall risk of bias; six trials were at high risk and the remaining ten had some concerns, mainly related to the randomization process and insufficient intervention-related reporting.*

*Very serious inconsistency was assigned when heterogeneity was extreme and clinically difficult to explain (for example, I² 88%–99% across several biomarker outcomes and 93% for length of hospital stay). Serious inconsistency was assigned to APACHE II because heterogeneity remained substantial (I²=72%) despite the small number of studies.*

*Serious indirectness was assigned to laboratory biomarkers and amylase outcomes because these are surrogate biochemical endpoints rather than direct patient-important outcomes.*

*Serious imprecision was assigned to APACHE II and IL-2 because the total sample size was small and the evidence base included only three trials. Very serious imprecision was assigned to adverse events because a reliable pooled effect estimate could not be reconstructed from the published reports.*

*Publication bias was considered strongly suspected across outcomes because all included trials were conducted in mainland China, were mostly small single-center studies, and almost uniformly reported favorable effects.*
