# Supplementary material for: Comparative efficacy and safety of Xuesaitong injection as adjuvant therapy for acute pancreatitis: a systematic review and meta-analysis
Source: Front Pharmacol. 2026 Jul 29;17:1875983. doi: 10.3389/fphar.2026.1875983 (PMC13462745; doi:10.3389/fphar.2026.1875983)
Supplement: Supplementary file 3 [file Table3.docx]

**Supplementary File 3. Manufacturer, specification, composition, and dosage of Xuesaitong injection in the included studies**

| **Study** | **Manufacturer, Approval Number, and Executive Standard** | **Composition** | **Specification and Daily Dose** |
| --- | --- | --- | --- |
| Liu 2004 | Kunming Pharmaceutical Group Co., Ltd.; Z20026438; YBZ00362021 | Panax notoginseng saponins | 400 mg/vial; 400 mg/day |
| Liu 2006 | Kunming Pharmaceutical Group Co., Ltd.; Z20026438; YBZ00362021 | Panax notoginseng saponins | 400 mg/vial; 400 mg/day |
| Niu 2008 | NR | NR | 10 mL: 50 mg/vial; 50 mg/day |
| Du 2009 | NR | NR | 400 mg/vial; 400 mg/day |
| Li 2016 | NR | NR | 400 mg/vial; 400 mg/day |
| Fang 2017 | Heilongjiang ZBD Pharmaceutical Co., Ltd.; Z23020787; WS3-B-3590-2001(Z)-2011 | Panax notoginseng saponins | 100 mg/vial; 400 mg/day |
| Wang 2018 | Heilongjiang ZBD Pharmaceutical Co., Ltd.; Z20026437; WS3-B-3590-2001(Z)-2011 | Panax notoginseng saponins | 100 mg/vial; 400 mg/day |
| Shi 2018 | Heilongjiang ZBD Pharmaceutical Co., Ltd.; Z24120165; WS3-B-3590-2001(Z)-2011 | Panax notoginseng saponins | 100 mg/vial; 400 mg/day |
| Zhang 2018 | Heilongjiang ZBD Pharmaceutical Co., Ltd.; Z23020787; WS3-B-3590-2001(Z)-2011 | Panax notoginseng saponins | 10 mL/vial; 40 mL/day |
| Zhang 2019 | NR | NR | 40 mL/day |
| Yu 2019 | NR | NR | 40 mL/day |
| Zhang 2020 | Lonch Group Wanrong Pharmaceutical Co., Ltd.; Z14020079; WS3-B-3590-2001(Z)-2011 | Panax notoginseng saponins | 400 mg/vial; 400 mg/day |
| Lin 2020 | Kunming Pharmaceutical Group Co., Ltd.; Z20026438; YBZ00362021 | Panax notoginseng saponins | 200 mg/vial; AP:200 mg/day, SAP:400 mg/day |
| Zou 2021 | Kunming Pharmaceutical Group Co., Ltd.; Z20026438; YBZ00362021 | Panax notoginseng saponins | 5 mL: 175 mg/vial; 400 mg/day |
| Song 2021 | Guangxi Wuzhou Pharmaceutical Group Co., Ltd.; Z20025652; WS3-B-3590-2001(Z)-2011 | Panax notoginseng saponins | 250 mg/vial; 500 mg/day |
| Liu 2024 | Kunming Pharmaceutical Group Co., Ltd.; Z20026438; YBZ00362021 | Panax notoginseng saponins | 400 mg/vial; 400 mg/day |

Abbreviation: NR, not reported.
