# Supplementary material for: Comparative efficacy and safety of Xuesaitong injection as adjuvant therapy for acute pancreatitis: a systematic review and meta-analysis
Source: Front Pharmacol. 2026 Jul 29;17:1875983. doi: 10.3389/fphar.2026.1875983 (PMC13462745; doi:10.3389/fphar.2026.1875983)
Supplement: Supplementary file 4 [file Table4.docx]

**Supplementary File 4. Definitions of Clinical response rate in each study**

| **Study** | **Definition of clinical response** |
| --- | --- |
| Du 2009 | Clinical symptoms and signs were relieved within 3 days and disappeared within 7 days, with laboratory test results returning to normal; or clinical symptoms and signs markedly improved within 7 days, with laboratory test results returning to normal; or clinical symptoms and signs improved within 7 days, with laboratory test results decreased but not completely returning to normal. |
| Fang 2017 | Serum and urinary amylase levels returned to normal, and clinical symptoms completely disappeared; or serum and urinary amylase levels nearly returned to normal, with improvement in clinical symptoms. |
| Wang 2018 | Clinical symptoms disappeared, and serum and urinary amylase levels as well as serum inflammatory factor levels returned to normal; or clinical symptoms markedly improved, and serum and urinary amylase levels as well as serum inflammatory factor levels nearly returned to normal. |
| Shi 2018 | Clinical symptoms basically disappeared, and serum and urinary amylase levels nearly returned to normal; or clinical symptoms improved, and serum and urinary amylase levels showed recovery. |
| Zhang 2018 | Clinical indicators returned to normal, and clinical symptoms disappeared; or clinical indicators nearly returned to normal, with marked improvement in clinical symptoms. |
| Zhang 2019 | Clinical symptoms completely disappeared, and serum and urinary amylase levels returned to normal; or clinical symptoms markedly improved, and serum and urinary amylase levels nearly returned to normal. |
| Yu 2019 | Clinical symptoms disappeared, and all laboratory indicators returned to normal; or clinical symptoms markedly improved, and all laboratory indicators nearly returned to normal. |
| Zhang 2020 | Clinical symptoms disappeared, and serum and urinary amylase levels returned to normal; or clinical symptoms markedly improved, and serum and urinary amylase levels nearly returned to normal. |
| Lin 2020 | Clinical response was defined as complete disappearance of clinical symptoms such as abdominal pain, no morphological changes in pancreatic tissue on abdominal CT, and normalization of serum and urinary amylase levels; near-complete disappearance of symptoms of symptoms, no obvious morphological changes on abdominal CT, and a reduction in serum and urinary amylase levels by >70%; or marked relief of symptoms, mild residual morphological changes in pancreatic tissue, and a reduction in serum and urinary amylase levels by 50%–70%. |
| Zou 2021 | Clinical symptoms completely disappeared; or clinical symptoms improved or were relieved. |
| Song 2021 | Symptoms disappeared or basically disappeared, with a symptom score reduction of ≥95%; or symptoms markedly improved, with a symptom score reduction of ≥70% but <95%; or symptoms improved, with a symptom score reduction of ≥30% but <70%. |
| Liu 2024 | Symptoms, including upper abdominal pain accompanied by nausea and vomiting, disappeared, and laboratory test results returned to normal; or symptoms markedly improved, and laboratory test results returned to normal; or symptoms were relieved, and laboratory test results decreased but did not reach the normal range. |
