# Supplementary material for: Comparative efficacy and safety of Xuesaitong injection as adjuvant therapy for acute pancreatitis: a systematic review and meta-analysis
Source: Front Pharmacol. 2026 Jul 29;17:1875983. doi: 10.3389/fphar.2026.1875983 (PMC13462745; doi:10.3389/fphar.2026.1875983)
Supplement: Supplementary file 5 [file Table5.docx]

**PRISMA 2020 Checklist**

Manuscript: Comparative efficacy and safety of Xuesaitong injection as adjuvant therapy for acute pancreatitis: a systematic review and meta-analysis

| **Section and Topic** | **Item #** | **Checklist item** |  |
| --- | --- | --- | --- |
| **TITLE** | | | |
| Title | 1 | Identify the report as a systematic review. | Title page. The title identifies the report as a systematic review and meta-analysis. |
| **ABSTRACT** | | | |
| Abstract | 2 | See the PRISMA 2020 for Abstracts checklist. | Abstract. The structured abstract reports background, methods, results, and conclusion; it also identifies the study as a systematic review and meta-analysis. |
| **INTRODUCTION** | | | |
| Rationale | 3 | Describe the rationale for the review in the context of existing knowledge. | Introduction, paragraphs 1-5. The rationale is described in relation to acute pancreatitis burden, current supportive management, and the pharmacological rationale for Xuesaitong injection. |
| Objectives | 4 | Provide an explicit statement of the objective(s) or question(s) the review addresses. | Introduction, final paragraph; Abstract, Methods/Objective statements. The review objective is to evaluate the efficacy and safety of Xuesaitong injection as adjuvant therapy for acute pancreatitis. |
| **METHODS** | | | |
| Eligibility criteria | 5 | Specify the inclusion and exclusion criteria for the review and how studies were grouped for the syntheses. | Methods 2.3, Eligibility criteria (PICO framework); Methods 2.7, Statistical analysis. Inclusion/exclusion criteria and outcome groupings for synthesis are specified. |
| Information sources | 6 | Specify all databases, registers, websites, organisations, reference lists and other sources searched or consulted to identify studies. Specify the date when each source was last searched or consulted. | Methods 2.4, Literature search. Databases searched: Cochrane Library, Embase, Web of Science, PubMed, CNKI, Wanfang, VIP, and CBM/Sinomed, from inception to 1 March 2026; reference lists of included studies and relevant reviews were also manually screened. |
| Search strategy | 7 | Present the full search strategies for all databases, registers and websites, including any filters and limits used. | Methods 2.4, Literature search; Supplementary File 1. Full database-specific search strategies are stated to be provided in Supplementary File 1. |
| Selection process | 8 | Specify the methods used to decide whether a study met the inclusion criteria of the review, including how many reviewers screened each record and each report retrieved, whether they worked independently, and if applicable, details of automation tools used in the process. | Methods 2.5, Screening and data extraction. Two reviewers independently screened titles/abstracts and full texts according to predefined criteria; disagreements were resolved by discussion or third-reviewer arbitration. |
| Data collection process | 9 | Specify the methods used to collect data from reports, including how many reviewers collected data from each report, whether they worked independently, any processes for obtaining or confirming data from study investigators, and if applicable, details of automation tools used in the process. | Methods 2.5, Screening and data extraction. Two reviewers independently extracted data using a pre-designed form, cross-checked the extracted information, and attempted to contact original authors when data were incomplete or ambiguous. |
| Data items | 10a | List and define all outcomes for which data were sought. Specify whether all results that were compatible with each outcome domain in each study were sought (e.g. for all measures, time points, analyses), and if not, the methods used to decide which results to collect. | Methods 2.3.4, Outcomes. Primary outcomes were clinical response rate and adverse events; secondary outcomes included length of hospital stay, APACHE II score, serum amylase, urinary amylase, CRP, IL-6, IL-2, TNF-alpha, and sICAM-1. The manuscript does not explicitly state whether all eligible time points or analyses within each outcome domain were sought; consider adding this if required. |
| Data items | 10b | List and define all other variables for which data were sought (e.g. participant and intervention characteristics, funding sources). Describe any assumptions made about any missing or unclear information. | Methods 2.5, Screening and data extraction; Results 3.2 and Table 2. Variables sought included first author, publication year, sample size, age, baseline characteristics, intervention/control regimens, dosage, treatment duration, raw outcome data, and methodological elements for risk-of-bias assessment. |
| Study risk of bias assessment | 11 | Specify the methods used to assess risk of bias in the included studies, including details of the tool(s) used, how many reviewers assessed each study and whether they worked independently, and if applicable, details of automation tools used in the process. | Methods 2.6, Risk of bias assessment. Two reviewers independently assessed risk of bias using Cochrane RoB 2 across five domains, with disagreements resolved by discussion or third-reviewer arbitration. |
| Effect measures | 12 | Specify for each outcome the effect measure(s) (e.g. risk ratio, mean difference) used in the synthesis or presentation of results. | Methods 2.7, Statistical analysis. RR with 95% CI was used for dichotomous outcomes; MD with 95% CI was used for continuous outcomes with consistent units; SMD was used when scales or units differed. |
| Synthesis methods | 13a | Describe the processes used to decide which studies were eligible for each synthesis (e.g. tabulating the study intervention characteristics and comparing against the planned groups for each synthesis (item #5)). | Methods 2.3, Eligibility criteria; Methods 2.7, Statistical analysis; Results 3.2 and Table 2. Studies were grouped by PICO eligibility, outcome availability, and treatment duration for subgroup analyses. |
| Synthesis methods | 13b | Describe any methods required to prepare the data for presentation or synthesis, such as handling of missing summary statistics, or data conversions. | Methods 2.5 and 2.7. Missing or ambiguous data were addressed by attempting to contact original authors; SMD was used when scales differed or units could not be standardized. Specific imputation/conversion rules for missing summary statistics are not fully detailed. |
| Synthesis methods | 13c | Describe any methods used to tabulate or visually display results of individual studies and syntheses. | Methods 2.7; Figures 3-9; Tables 2-5. Forest plots, funnel plot, and structured tables were used to present study characteristics, pooled estimates, sensitivity analysis, subgroup analysis, and adverse events. |
| Synthesis methods | 13d | Describe any methods used to synthesize results and provide a rationale for the choice(s). If meta-analysis was performed, describe the model(s), method(s) to identify the presence and extent of statistical heterogeneity, and software package(s) used. | Methods 2.7. Random-effects meta-analysis was used for all outcomes. Mantel-Haenszel method was used for dichotomous outcomes and inverse-variance method for continuous outcomes. Heterogeneity was assessed using Cochran Q and I-squared. RevMan 5.4 was used for main analyses and graphical outputs; R 4.3.3 was used for supplementary tests. |
| Synthesis methods | 13e | Describe any methods used to explore possible causes of heterogeneity among study results (e.g. subgroup analysis, meta-regression). | Methods 2.7; Results 3.11 and Table 4. Prespecified subgroup analyses by treatment duration (<=7 days vs >7 days) were used to explore heterogeneity. |
| Synthesis methods | 13f | Describe any sensitivity analyses conducted to assess robustness of the synthesized results. | Methods 2.7; Results 3.10 and Table 3. Sensitivity analyses included leave-one-out analyses and exclusion of studies judged to have high overall risk of bias. |
| Reporting bias assessment | 14 | Describe any methods used to assess risk of bias due to missing results in a synthesis (arising from reporting biases). | Methods 2.7, Statistical analysis. Funnel plots with Harbord or Egger tests were planned for outcomes with 10 or more studies to assess reporting bias/small-study effects. |
| Certainty assessment | 15 | Describe any methods used to assess certainty (or confidence) in the body of evidence for an outcome. | Methods 2.8, Quality of evidence appraisal. GRADE was used to assess certainty of evidence across risk of bias, inconsistency, indirectness, imprecision, and publication bias. |
| **RESULTS** | | | |
| Study selection | 16a | Describe the results of the search and selection process, from the number of records identified in the search to the number of studies included in the review, ideally using a flow diagram. | Results 3.1 and Figure 1. The manuscript reports 135 records identified, 108 duplicates removed, 27 records screened, 21 full-text reports assessed, and 16 studies included in qualitative and quantitative syntheses. |
| Study selection | 16b | Cite studies that might appear to meet the inclusion criteria, but which were excluded, and explain why they were excluded. | Results 3.1 and Figure 1. Excluded records and full-text exclusions are summarized by reason. |
| Study characteristics | 17 | Cite each included study and present its characteristics. | Results 3.2 and Table 2. All 16 included studies are cited and their sample size, demographics, Xuesaitong regimen, control regimen, outcomes, and treatment duration are presented. |
| Risk of bias in studies | 18 | Present assessments of risk of bias for each included study. | Results 3.3 and Figure 2. RoB 2 assessments are summarized by domain and overall judgment; six studies were rated high risk and ten as some concerns. |
| Results of individual studies | 19 | For all outcomes, present, for each study: (a) summary statistics for each group (where appropriate) and (b) an effect estimate and its precision (e.g. confidence/credible interval), ideally using structured tables or plots. | Results 3.4-3.9; Figures 3-8; Table 5. Individual study results and pooled effects are primarily presented in forest plots and the adverse-event table. Ensure each forest plot contains study-level summary statistics and effect estimates. |
| Results of syntheses | 20a | For each synthesis, briefly summarise the characteristics and risk of bias among contributing studies. | Results 3.3-3.9. Contributing studies are described for each outcome, and overall risk-of-bias findings are reported. Partially reported: synthesis-specific risk-of-bias summaries are not separately repeated for each outcome. |
| Results of syntheses | 20b | Present results of all statistical syntheses conducted. If meta-analysis was done, present for each the summary estimate and its precision (e.g. confidence/credible interval) and measures of statistical heterogeneity. If comparing groups, describe the direction of the effect. | Results 3.4-3.9; Figures 3-8. All meta-analytic syntheses report pooled estimates with 95% CIs, P values, heterogeneity statistics, and direction of effect. |
| Results of syntheses | 20c | Present results of all investigations of possible causes of heterogeneity among study results. | Results 3.11 and Table 4. Subgroup analyses by treatment duration are reported, including subgroup estimates, heterogeneity, and interaction P values. |
| Results of syntheses | 20d | Present results of all sensitivity analyses conducted to assess the robustness of the synthesized results. | Results 3.10 and Table 3. Sensitivity analyses are reported, including analyses excluding high-risk studies and selected leave-one-out results. |
| Reporting biases | 21 | Present assessments of risk of bias due to missing results (arising from reporting biases) for each synthesis assessed. | Results 3.13 and Figure 9. Reporting bias was assessed for clinical response rate using a funnel plot and Harbord test; no significant small-study effect was detected. Other outcomes were not assessed because fewer than 10 studies contributed. |
| Certainty of evidence | 22 | Present assessments of certainty (or confidence) in the body of evidence for each outcome assessed. | Results 3.14; Supplementary File 2. GRADE certainty is summarized for each outcome; clinical response rate was rated low, and the remaining outcomes were rated very low. |
| **DISCUSSION** | | | |
| Discussion | 23a | Provide a general interpretation of the results in the context of other evidence. | Discussion, paragraphs 1-2. The main findings are interpreted in the context of acute pancreatitis pathophysiology and pharmacological evidence for Panax notoginseng saponins/Xuesaitong injection. |
| Discussion | 23b | Discuss any limitations of the evidence included in the review. | Discussion, limitations paragraph. The manuscript discusses limitations in the included evidence, including limited methodological quality, high risk of bias, heterogeneity, small numbers of studies for some outcomes, and restriction to studies conducted in China. |
| Discussion | 23c | Discuss any limitations of the review processes used. | Discussion, limitations paragraph. Partially reported: limitations of the review process itself, such as possible database/language limitations, inability to obtain missing data, or reliance on published aggregate data, are not fully separated from limitations of the evidence. |
| Discussion | 23d | Discuss implications of the results for practice, policy, and future research. | Discussion, safety/limitations paragraphs; Conclusion. The manuscript discusses cautious clinical interpretation, need for standardized use and monitoring, and the need for multicenter, large-sample, double-blind RCTs. |
| **OTHER INFORMATION** | | | |
| Registration and protocol | 24a | Provide registration information for the review, including register name and registration number, or state that the review was not registered. | Methods 2.2, Study profile. The review was prospectively registered in PROSPERO, registration number CRD420261335764. |
| Registration and protocol | 24b | Indicate where the review protocol can be accessed, or state that a protocol was not prepared. | Methods 2.2, Study profile. The protocol can be accessed through PROSPERO using registration number CRD420261335764. Consider adding a direct URL if required by the journal. |
| Registration and protocol | 24c | Describe and explain any amendments to information provided at registration or in the protocol. | None |
| Support | 25 | Describe sources of financial or non-financial support for the review, and the role of the funders or sponsors in the review. | Funding section. Funding sources are reported. |
| Competing interests | 26 | Declare any competing interests of review authors. | Conflict of interest section. The authors declare no commercial or financial relationships that could be construed as a potential conflict of interest. |
| Availability of data, code and other materials | 27 | Report which of the following are publicly available and where they can be found: template data collection forms; data extracted from included studies; data used for all analyses; analytic code; any other materials used in the review. | Data availability statement; Supplementary Material. The manuscript states that original contributions are included in the article/Supplementary Material. |
